# Synergistic Anti-Tumor Efficacy of Modified FOLFIRINOX and NK Cell Therapy in Pancreatic Ductal Adenocarcinoma

**DOI:** 10.3390/cancers17172785

**Published:** 2025-08-26

**Authors:** Hye-Seong Park, Jun Eul Hwang, Je-Jung Lee, Woo Kyun Bae

**Affiliations:** 1 Research and Development Center, VaxCell-Bio Therapeutics, Hwasun 58141, Republic of Korea; hyeseong523@gmail.com; 2 Research Center for Cancer Immunotherapy, Chonnam National University Hwasun Hospital, Hwasun 58128, Republic of Korea; 3 Division of Hematology-Oncology, Department of Internal Medicine, Chonnam National University Medical School and Hwasun Hospital, Jeonnam 58128, Republic of Korea; hjunyl@naver.com; 4 Immunotherapy Innovation Center, Chonnam National University Medical School and Hwasun Hospital, Jeonnam 58128, Republic of Korea

**Keywords:** pancreatic ductal adenocarcinoma (PDAC), natural killer (NK) cell, modified FOLFIRINOX (mFOLFIRINOX), NK-cell-activating ligands, apoptosis-inducing receptors

## Abstract

**Simple Summary:**

Pancreatic ductal adenocarcinoma (PDAC) remains one of the most lethal malignancies with limited response to current treatments. Natural killer (NK)-cell-based immunotherapy has shown promise, but tumor-induced immune evasion reduces its efficacy. This study aimed to investigate whether combining modified FOLFIRINOX (mFOLFIRINOX) chemotherapy with NK cell adoptive therapy could improve therapeutic outcomes. We demonstrated that mFOLFIRINOX enhances the expression of NK-cell-activating ligands such as NKG2D ligands on PDAC cells, making them more susceptible to NK-cell-mediated cytotoxicity. In vitro co-culture experiments and in vivo xenograft mouse models confirmed synergistic antitumor effects, leading to reduced tumor growth and improved survival. These findings suggest that mFOLFIRINOX can sensitize tumors to NK cell therapy, providing a potential strategy to overcome immune resistance in PDAC.

**Abstract:**

Background/Objectives: Pancreatic ductal adenocarcinoma (PDAC) presents a formidable challenge in oncology due to its aggressive progression, propensity for early metastasis, and resistance to conventional therapies. The development of effective and less toxic treatments is crucial for improving the prognosis of PDAC. We aimed to investigate the synergistic antitumor potential of modified FOLFIRINOX (mFOLFIRINOX) combined with natural killer (NK) cell therapy in PDAC models. Methods: We evaluated changes in NK-cell-activating ligands and apoptosis-inducing receptor expression after mFOLFIRINOX treatment both in vitro and in vivo. Subsequently, NK cells were administered to mFOLFIRINOX-pre-treated PDAC cells to assess NK cell cytotoxicity, immune responses, and tumor progression both in vitro and in vivo mouse models. Results: Treatment with mFOLFIRINOX led to the significant upregulation of NK-cell-activating ligands and apoptosis-inducing receptors across the PDAC cell lines and tumor cells collected in vivo, thereby enhancing their susceptibility to NK-cell-mediated cytotoxicity. In comparison with either treatment alone, mFOLFIRINOX and NK cell combination therapy resulted in enhanced cytolysis in all cell lines. In vivo studies demonstrated that combination therapy substantially inhibited tumor growth and prolonged survival in a mouse model. Conclusions: mFOLFIRINOX combined with NK cell therapy demonstrates enhanced antitumor activity against PDAC, potentially improving clinical outcomes. These findings highlight the need for continued research to optimize this combination strategy for clinical utility.

## 1. Introduction

Pancreatic cancer, particularly pancreatic ductal adenocarcinoma (PDAC), which constitutes over 85% of all pancreatic malignancies, presents substantial challenges in the field of oncology. Ranked as the seventh leading cause of oncological mortality worldwide for both genders, its prognosis is dismal. Projections indicate that by 2030, PDAC will rise to the second leading cause of cancer-related mortality in the United States [1,2,3]. PDAC is distinguished by an aggressive clinical course, a low likelihood of surgical resectability due to its propensity for early metastasis, high rates of recurrence, and relative resistance to current therapeutic modalities [2,3,4,5]. Despite advancements in oncological therapeutics, the 5-year survival rate for pancreatic cancer has only incrementally improved, remaining at a low rate of less than 9% [3]. The predominantly asymptomatic development of PDAC, along with a deficiency in reliable early detection biomarkers, usually leads to delayed diagnoses at stages when the disease has already progressed to a locally advanced or metastatic stage, thus reducing the potential for effective treatment [2,3].

The treatment of PDAC typically involves systemic chemotherapy, with radiation therapy often utilized in cases of borderline resectable or locally advanced disease, while its use in metastatic stages is generally limited to palliative purposes. The chemotherapeutic regimen FOLFIRINOX comprising fluorouracil, leucovorin, irinotecan, and oxaliplatin, as well as a combination of gemcitabine and nab-paclitaxel, currently forms the basis of first-line therapeutic strategies [4]. Nevertheless, these conventional treatments often result in suboptimal clinical outcomes. This is evident in the high rates of recurrence and distant organ metastasis, which characterize the inherently aggressive behavior of PDAC [2,4,6,7].

Immunotherapy represents a major advancement in cancer therapeutics, introducing novel modalities when traditional treatments have reached their limitations [7]. This innovative approach includes a spectrum of strategies designed to strengthen or activate the immune system’s inherent cancer-fighting capability [8]. Notably, checkpoint inhibitors such as pembrolizumab and nivolumab have revolutionized the field by disengaging immune checkpoints, thereby enhancing immune cell attack against neoplastic cells. Concurrently, adoptive cell transfer (ACT) has emerged as a novel technique where a patient’s immune constituents, including NK cells, T cells, and cells expressing chimeric antigen receptor (CAR), are engineered or amplified ex vivo to augment their tumoricidal potency [7,9].

Natural killer (NK) cell therapy is an indispensable immunotherapy modality, in which the intrinsic cytotoxic capabilities of NK cells are exploited to target and eliminate cancer cells without the need for prior sensitization. As key elements of the innate immune system, NK cells play a critical role in the rapid response to neoplastic cells. Their capacity for swift and decisive cytotoxic action against tumor cells, orchestrated by a complex interplay of inhibitory and activating signals, positions them as potential candidates in the arsenal of cancer therapeutics [10].

Interestingly, the components of FOLFIRINOX have been observed to induce the upregulation of NK cell ligands on tumor cells [11,12,13,14,15], a phenomenon that is also noted with other chemotherapeutic agents. This upregulation also extends to FAS and death receptors, which are crucial for the apoptotic pathways of cancer cells [16,17,18]. Importantly, several studies have shown that pre-treatment with standard chemotherapeutic agents—such as 5-fluorouracil, oxaliplatin, and irinotecan—can enhance the expression of NK-cell-activating ligands and death receptors on tumor cells, leading to increased NK cell recognition and cytotoxicity both in vitro and in vivo [11,12,13,14,19]. A similar immunogenic modulation effect has also been reported with gemcitabine in pancreatic cancer models, where increased expression of NKG2D ligands sensitized tumor cells to NK-cell-mediated killing [18].

These findings suggest that chemotherapy may sensitize tumor cells to NK-cell-mediated cytotoxicity, thereby supporting the rationale for combining cytotoxic chemotherapy with NK cell immunotherapy. We hypothesize that the upregulation of ligands and receptors induced by FOLFIRINOX could make tumor cells more susceptible to NK-cell-mediated cytotoxicity. The potential synergy between chemotherapy and NK cell therapy could significantly amplify anti-tumor effects in PDAC.

In our study, we focused on evaluating the combined effects of NK cell therapy with modified FOLFIRINOX (mFOLFIRINOX) in the treatment of PDAC. mFOLFIRINOX alters the standard regimen by reducing the dose and omitting the 5-fluorouracil intravenous bolus, thereby mitigating toxicity with comparable anti-tumor efficacy [20,21,22]. We aimed to examine how mFOLFIRINOX influences the expression of NK-cell-activating ligands and death receptors in PDAC cells and to assess the subsequent enhancement in NK-cell-mediated cytotoxicity using both in vitro approaches and an in vivo NSG mouse model. A combination of mFOLFIRINOX and NK cell therapy could potentially revolutionize the treatment landscape for PDAC, offering a more effective treatment paradigm.

## 2. Materials and Methods

### 2.1. Ethical Approval

All methods were conducted in accordance with relevant guidelines and regulations.

#### 2.1.1. Animal Experiments

All procedures involving animal experiments were approved by the Institutional Animal Care and Use Committee (IACUC) of Chonnam National University (IACUC approval No. CNU IACUC-H-2023-51). All methods involving animals were performed in accordance with the ARRIVE guidelines to ensure thorough and transparent reporting of animal research.

#### 2.1.2. Human Subjects

Human blood samples were obtained following the protocol approved by the institutional review board (IRB) of Chonnam National University Hwasun Hospital (IRB approval No. CNUHH-2021-029). Informed consent was obtained from all healthy volunteers for the collection and use of their blood samples.

### 2.2. Cell Lines and Drugs

The human PDAC cell lines CFPAC-1 and PANC-1 were procured from the American Type Culture Collection (ATCC, Manassas, VA, USA). The AsPC-1 cell line, which expresses luciferase (AsPC-1/Luc), was sourced from the Japanese Collection of Research Bioresources Cell Bank (JCRB, Osaka, Japan). Additionally, the human chronic myelogenous leukemia cell line K562 was obtained from the ATCC. These cell lines were maintained in RPMI1640 medium, enriched with 10% (*v*/*v*) fetal bovine serum (FBS; Gibco, Carlsbad, CA, USA) and 1% (*v*/*v*) penicillin/streptomycin (P/S; Gibco, Carlsbad, CA, USA), and incubated at 37 °C in a humidified atmosphere containing 5% CO_2_.

For the mFOLFIRINOX regimen, the following agents were procured: oxaliplatin (Belloxa liquid^®^) from Chong Kun Dang (Seoul, Republic of Korea), irinotecan (Campto^®^) from Boryung Pharmaceutical (Seoul, Republic of Korea), 5-fluorouracil (JW 5FU^®^) from JW Pharmaceutical (Gwacheon, Republic of Korea), and leucovorin (Pfizer Leucovorin^®^) from Pfizer (New York, NY, USA). The drugs were utilized at the following concentrations for in vitro and in vivo experiments: oxaliplatin at 2.14 μM, irinotecan at 2.56 μM, 5-fluorouracil at 92.2 μM, and leucovorin at 8.45 μM for in vitro applications; oxaliplatin at 1.5 mg/kg, irinotecan at 2 mg/kg, 5-fluorouracil at 4 mg/kg, and leucovorin at 2 mg/kg for in vivo administration.

### 2.3. NK Cell Culture

NK cells were grown using our previously established K562 feeder cells [23]. Peripheral blood mononuclear cells (PBMCs) were initially isolated through density gradient centrifugation using Lymphoprep™ (Axis-Shield, Oslo, Norway). These PBMCs were then cultured and expanded with 100 Gy gamma-irradiated K562 cells in RPMI1640 medium enriched with 10% FBS, 1% P/S, and 4 mM L-glutamine (Gibco, Carlsbad, CA, USA). The culture was maintained in the presence of 10 U/mL recombinant human IL-2 (PeproTech, Cranbury, NJ, USA), and the cells were replenished with fresh medium and cytokines every 2–3 days. At the initiation of NK cell culture, additional cytokines, specifically 5 ng/mL recombinant human IL-21 (PeproTech, Cranbury, NJ, USA) and 10 ng/mL recombinant human 4-1BB ligand, were incorporated. After 7 days, the IL-2 concentration was increased to 100 U/mL, and 5 ng/mL recombinant human IL-15 (PeproTech, Cranbury, NJ, USA) was introduced. On day 14, the resulting expanded NK cells were harvested for subsequent in vitro and in vivo experimentation.

### 2.4. Flow Cytometry for NK Cell Phenotype and Ligand Expression

The purity and phenotype of NK cells were determined by flow cytometry. The cells were stained with a panel of fluorochrome-conjugated monoclonal antibodies, including Live/Dead Ghost dye, CD45, CD3, CD56, CD16, CD69, CD94, NKp30, NKp44, NKp46, NKG2D, NKG2A, CD158a, and CD158b (Table 1). To confirm the functional integrity of expanded NK cells prior to experimental use, a cytotoxicity assay was performed as part of quality control, following a previously established method [24]. For this, NK cells were labeled with carboxyfluorescein diacetate succinimidyl ester (CFSE; Life Technologies, Carlsbad, CA, USA) for 10 min. K562 cells were co-cultured with NK cells at an E:T ratio of 4:1 at 37 °C for 4 h in a 5% CO_2_ incubator. Subsequently, 1 μL of propidium iodide (Life Technologies, Carlsbad, CA, USA) was added. Tumor cells, with or without mFOLFIRINOX pre-treatment, were dissociated using StemPro™ Accutase™ (Thermo Fisher Scientific, Waltham, MA, USA) and stained with fluorochrome-conjugated monoclonal antibodies: ULBP-1, ULBP-2,5,6, ULBP-3, ULBP-4, MIC A/B, DR4, DR5, FAS, B7-H6, CD112, CD155, and Live/Dead Ghost dye (Table 1). Live/Dead staining was performed for 20 min, followed by antibody staining for 20 min. Data were acquired using the Attune NxT Flow Cytometer (Thermo Fisher Scientific, Waltham, MA, USA), and analysis was performed using FlowJo v10.10 (BD Biosciences, San Jose, CA, USA).

### 2.5. Cytotoxicity Assessment by Real-Time Cell Analysis (RTCA)

The cytotoxic activity of NK cells against PDAC cell lines, pre-treated with mFOLFIRINOX, was evaluated using a xCELLigence RTCA system (Agilent Technologies, Santa Clara, CA, USA). Initially, 1 × 10^4^ tumor cells/well were seeded in E-plate 96 (Agilent Technologies, Santa Clara, CA, USA) and allowed to adhere for 24 h. Then, these cells were treated with mFOLFIRINOX. Following 24 h of mFOLFIRINOX exposure, NK cells were introduced to the wells for co-culture at various E:T ratios, with an emphasis on an 8:1 ratio. Cell index (CI) values, indicative of cell viability and proliferation, were recorded at 30 min intervals over a 96 h period post-NK cell addition. The CI values were normalized to the time point immediately before the addition of the NK cells. To evaluate the cytotoxicity, the normalized cell index values were then converted into cytotoxicity–time curves. The resulting normalized cell index–time and cytotoxicity–time curves were plotted and analyzed using the RTCA Software Pro (https://www.agilent.com/ko-kr/product/cell-analysis/cell-analysis-software/cell-analysis-instrument-software/rtca-software-pro-741236 (accessed on 22 August 2025), Agilent Technologies, Santa Clara, CA, USA).

### 2.6. NK Cell Degranulation Assay and Intracellular Staining

To evaluate the functional response of NK cells to pancreatic cancer cell lines (AsPC-1, CFPAC-1, and PANC-1) pre-treated with mFOLFIRINOX, we co-cultured NK cells with these cancer cells at E:T ratios of 2:1 for 5 h in the presence of CD107a antibody. After co-culture for 1 h, we added the Protein Transport Inhibitor Cocktail (eBioscience, San Diego, CA, USA) to halt protein transport. Following co-culture, the cells were stained for 20 min using Live/Dead Ghost dye, CD45, CD3, CD56. Intracellular cytokine staining (ICS) was then performed with the Intracellular Fixation & Permeabilization Buffer Set (eBioscience, San Diego, CA, USA). The cells were further stained with specific antibodies targeting key effector molecules (IFN-γ, TNF-α, perforin, and granzyme B) for 40 min. The antibodies used to assess degranulation, and the expression of cytotoxic molecules and cytokines are listed in Table 1. Flow cytometry was conducted to quantify the expression of these effector molecules in NK cells, with data analysis performed using FlowJo software (BD Biosciences, San Jose, CA, USA).

### 2.7. Enzyme-Linked Immunosorbent Assay (ELISA)

PDAC cell lines (AsPC-1, CFPAC-1, and PANC-1) (1 × 10^5^ cells/well) were either pre-treated with mFOLFIRINOX or left untreated before being used as target cells. NK cells were then co-cultured with these target cells at an E:T ratio of 2:1 in 24-well plates (SPL, Pocheon, Republic of Korea) in a 5% CO_2_ incubator at 37 °C for 24 h. Supernatants were subsequently harvested to measure the concentrations of IFN-γ and TNF-α using the OptEIA ELISA kit (BD Biosciences, San Jose, CA, USA) following the manufacturer’s protocol. NK cells alone served as the negative control for the assay. Additionally, IFN-γ levels in the serum of mice were qualified using the same OptEIA ELISA kit (BD Biosciences, San Jose, CA, USA).

### 2.8. In Vivo Xenograft Model

Female NOD/SCID IL-2Rγ^null^ (NSG) mice (4–6 weeks old) (Jackson Laboratory, Bar Harbor, ME, USA) were raised under specific pathogen-free (SPF) conditions. To establish a PDAC xenograft model, we intraperitoneally injected human AsPC-1/Luc cells (1 × 10^6^ per mouse) into 6–8-week-old female NSG mice.

On day 0, the mice were intraperitoneally injected with 1 × 10^6^ AsPC-1/Luc cells. Three days post-tumor inoculation, the mice were divided into the following treatment groups: no treatment (PBS control), mFOLFIRINOX, NK cells (NK), and mFOLFIRINOX combined with NK cells (mFOLFIRINOX+NK). The mice were treated with mFOLFIRINOX or NK cells via intraperitoneal injection. The administration of mFOLFIRINOX and NK cells commenced 3 days following the xenograft of AsPC-1/Luc cells and was conducted over three cycles at 1-week intervals. The mFOLFIRINOX regimen involved the intraperitoneal injection of a drug combination on the first day of each cycle, comprising oxaliplatin (1.5 mg/kg), irinotecan (2 mg/kg), 5-fluorouracil (4 mg/kg), and leucovorin (2 mg/kg), followed by the intraperitoneal injection of 5-fluorouracil alone (4 mg/kg) on the subsequent day. NK cells (5 × 10^6^/mouse) were administered intraperitoneally for 2 consecutive days, starting concurrently with 5-fluorouracil.

To evaluate the effects of in vivo mFOLFIRINOX treatment on ligand expression in AsPC-1/Luc cells, flow cytometry was conducted to assess changes in ligand expression in the PDAC xenograft model. This analysis was performed on mice in both the PBS control group and the mFOLFIRINOX group. On days 12 and 19 post-tumor implantation, 3 mice from each group were euthanized, and their intraperitoneal cells were harvested by peritoneal lavage for flow cytometric analysis of NK-cell-activating ligand expression, following a previously established protocol [25].

Tumor growth in the mice was monitored weekly by bioluminescence imaging (BLI) with the Night Owl System (Berthold Technologies, Bad Wildbad, Germany). The mice received an intraperitoneal injection of D-luciferin (150 mg/kg/mouse; Perkin Elmer, Waltham, MA, USA) 10 min prior to imaging. Serum IFN-γ levels were assessed using the OptEIA ELISA kit (BD Biosciences, San Jose, CA, USA). The phenotype of NK cells within the tumor microenvironment (TME) was evaluated by flow cytometry after harvesting intraperitoneal cells and performing phenotypic surface staining.

Humane endpoints for euthanasia were established in compliance with the AVMA Guidelines for the Euthanasia of Animals (2020 Edition). Mice were monitored daily for signs of severe distress, pain, or suffering. Criteria for euthanasia included cachexia, severe clinical signs such as labored breathing, severe lethargy, or immobility, and moribund condition (i.e., near death, unresponsive to stimuli, or severe hypothermia).

Additionally, euthanasia was performed by a licensed veterinarian using a gradual-fill method of Co_2_ inhalation in a dedicated euthanasia chamber with a regulated flow rate of 30–70% of the chamber volume per minute, as recommended by the AVMA guidelines. The animals were continuously monitored for cessation of respiration and lack of response to physical stimuli. Following euthanasia, cervical dislocation was performed as the secondary physical confirmation of death to ensure humane practice.

### 2.9. Statistical Analysis

Data were analyzed using GraphPad Prism 10 software (GraphPad Software Inc., San Diego, CA, USA). Statistical significance was determined by Student’s *t*-test or one-way ANOVA. A log-rank test was performed on survival data. Data are presented as the mean ± standard deviation (SD) or standard error of the mean (SEM). Differences were considered statistically significant at * *p* < 0.05, ** *p* < 0.01, *** *p* < 0.001, and **** *p* < 0.0001.

## 3. Results

### 3.1. Effects of mFOLFIRINOX Pre-Treatment on NK-Cell-Activating Ligands and Apoptosis-Inducing Receptors in PDAC Cell Lines

**Figure 1:**
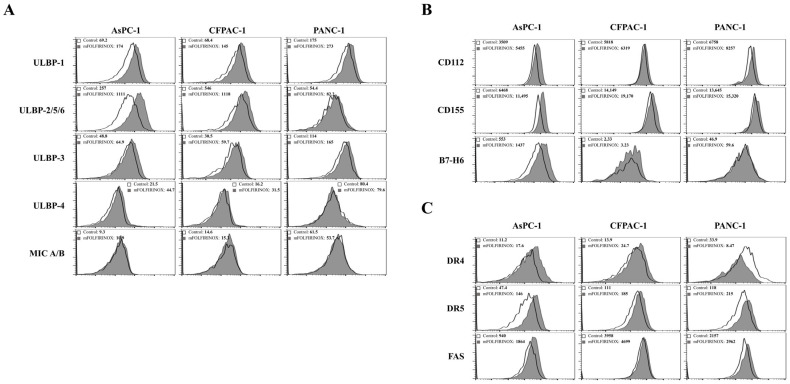
Effects of mFOLFIRINOX on the expression of NK-cell-activating ligands and apoptosis-inducing receptors in PDAC cells in vitro. PDAC cells (AsPC-1, CFPAC-1, and PANC-1) were treated with mFOLFIRINOX for 24 h. After 24 h, the expression of NKG2D ligands (**A**), DNAM-1 and NKp30 (**B**), and death receptor 4, 5 and FAS (**C**) was assessed by flow cytometry. Data are presented as histogram, and the mean fluorescence intensity (MFI) was calculated to compare the expression level between untreated and mFOLFIRINOX-treated cancer cells.

To assess the synergy between NK cells and mFOLFIRINOX in PDAC treatment, we examined the expression of NK-cell-activating ligands and apoptosis-inducing receptors in PDAC cell lines (AsPC-1, CFPAC-1, and PANC-1) following mFOLFIRINOX treatment for 24 h. The detailed gating strategy used for flow cytometric analysis is presented in Figure S1, and the expression data are shown in Figure 1. Post-treatment analysis revealed the upregulation of the NKG2D ligand ULBP-1 across all cell lines. Additionally, AsPC-1 and CFPAC-1 cells exhibited increased ULBP-2/5/6 expression, whereas PANC-1 cells showed minimal changes (Figure 1A). The DNAM-1 ligands CD112 and CD155 also demonstrated increased expression across the cell lines. A marked increase in B7-H6 expression, a ligand for NKp30, was observed predominantly in AsPC-1 cells (Figure 1B).

Furthermore, we evaluated the expression of the apoptosis-inducing receptors DR4, DR5, and FAS, which interact with TRAIL, TRAIL, and FAS-L, respectively. Although the expression levels of DR5 and FAS were elevated in all cell lines, DR4 remained unchanged (Figure 1C). These findings suggest that mFOLFIRINOX treatment could enhance NK cell activation against PDAC cells, underscoring the therapeutic potential of combining mFOLFIRINOX with NK cell therapy to improve anti-cancer efficacy.

### 3.2. Cytotoxic Efficacy of mFOLFIRINOX and NK Cell Combination Therapy Against PDAC Cell Lines

**Figure 2:**
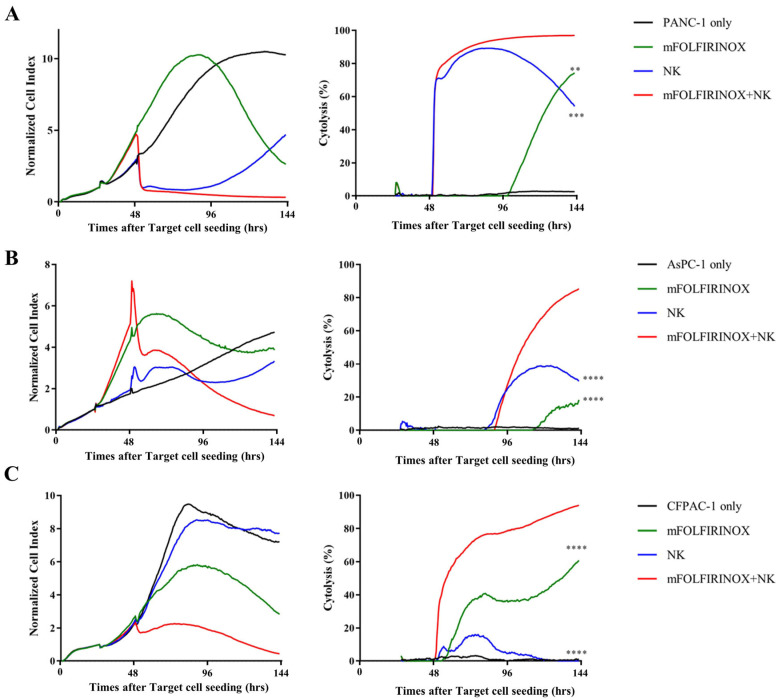
Cytotoxicity of mFOLFIRINOX and NK cell combination therapy against PDAC cells. PDAC cells were observed over 144 h with treatments at specific time points: cancer cell seeding (0 h), mFOLFIRINOX treatment (24 h), and NK cell treatment (48 h). Cancer cell growth and cytotoxicity were measured as normalized cell index and cytolysis (%), respectively. The effects of treatment on cancer cell growth and cytotoxicity against PANC-1 (**A**), AsPC-1 (**B**), and CFPAC-1 (**C**) cells were assessed by Real-Time Cell Analysis (RTCA). The groups evaluated were as follows: untreated tumor cells (Target cell only, black), mFOLFIRINOX monotherapy (mFOLFIRINOX, green), NK monotherapy (NK, blue), and mFOLFIRINOX and NK cell combination therapy (mFOLFIRINOX+NK, red). The statistical significance of cytotoxicity was determined at the endpoint (144 h) by one-way ANOVA, with each treatment group compared to the mFOLFIRINOX+NK group. *p* < 0.01 (**), *p* < 0.001 (***), *p* < 0.0001 (****).

We evaluated the cytotoxic efficacy of mFOLFIRINOX and NK cell combination therapy (mFOLFIRINOX+NK) against PDAC cell lines (PANC-1, AsPC-1, and CFPAC-1). Notably, all cell lines demonstrated enhanced cytolysis when subjected to combination therapy, with a marked improvement in cell lysis compared with the effects observed with either treatment alone (Figure 2). Although mFOLFIRINOX treatment exhibited cytotoxicity across all cell lines, the onset of cell lysis was comparatively slower and less pronounced than that following combination therapy. Although NK cell monotherapy was similar to combination therapy in cytolysis initiation, it resulted in significantly less cell destruction (Figures S2 and S3).

PANC-1 cells, which initially responded well to NK cell monotherapy, exhibited reduced cytolytic efficacy over time, with a cytolysis rate of 54.2% at the endpoint (144 h), indicating partial regrowth and emerging resistance to NK-cell-mediated killing, in contrast to the sustained high cytolysis rate of 96.9% observed in the mFOLFIRINOX+NK group (Figure 2A). AsPC-1 cells showed moderate resistance, with cytotoxicity levels remaining above 85% in the mFOLFIRINOX+NK group compared with the reduced efficacy in the NK group (Figure 2B). CFPAC-1 cells, which initially demonstrated high resistance to NK cells, showed a marked increase in sensitivity following mFOLFIRINOX pre-treatment, achieving 93.9% cytotoxicity in the mFOLFIRINOX+NK group (Figure 2C).

These findings demonstrated that combining mFOLFIRINOX with NK cell therapy could significantly enhance cytotoxicity against pancreatic cancer cell lines, effectively overcoming the inherent resistance observed in various cell lines. Therefore, this combination therapy provides a potentially improved therapeutic approach for treating PDAC.

### 3.3. Anti-Cancer Immune Response of NK Cells Following mFOLFIRINOX Pre-Treatment of PDAC Cell Line

To investigate the immune response of NK cells following mFOLFIRINOX pre-treatment of PDAC cell lines, we conducted a comprehensive analysis that included NK cell degranulation, the intracellular expression of cytotoxic molecules, and cytokine secretion (Figure S4).

**Figure 3:**
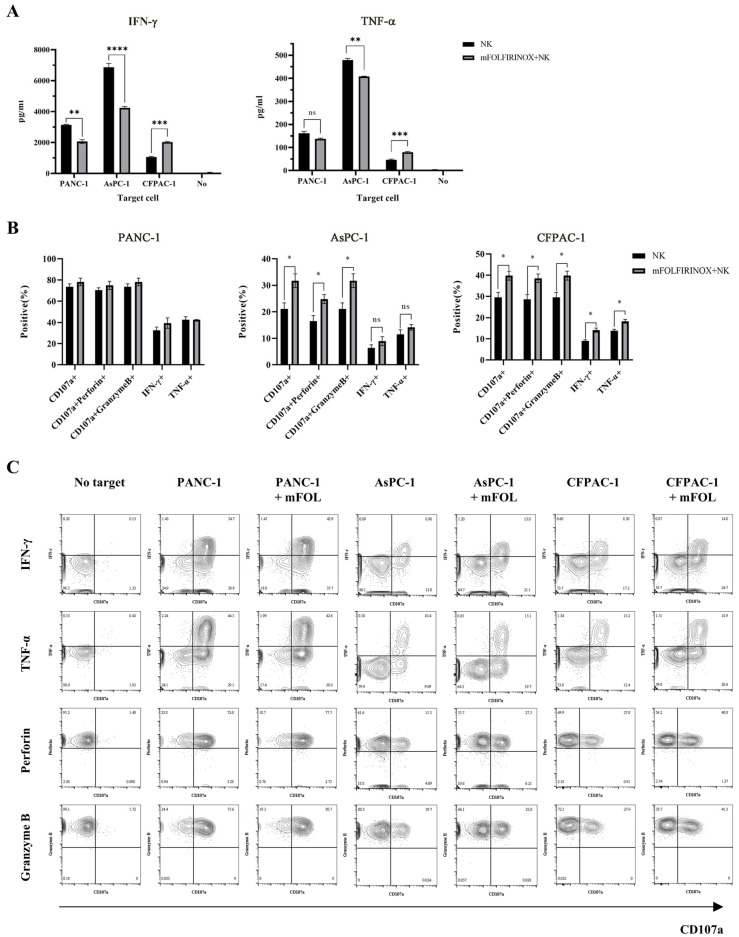
Immunological analysis of the anti-cancer efficacy of mFOLFIRINOX and NK cell combination therapy against PDAC cells. Following 24 h of pre-treatment with mFOLFIRINOX, pancreatic cancer cells were co-cultured with NK cells. Supernatants and NK cells were then collected for analysis. The concentrations of INF-γ and TNF-α in the supernatants were measured by ELISA (**A**). Degranulation capability was assessed based on the expression of CD107a. The expression levels of perforin, granzyme B, INF-γ, and TNF-α were analyzed in live CD45^+^CD3^−^CD56^+^ cells (**B**). Representative flow cytometry plots corresponding to the bar graphs in Figure 3B (**C**). Plots are arranged horizontally by experimental group (No target, PANC-1, PANC-1+mFOL, AsPC-1, AsPC-1+mFOL, CFPAC-1, CFPAC-1+mFOL) and vertically by marker (IFN-γ, TNF-α, perforin, granzyme B). ‘mFOL’ indicated co-culture with target cells pre-treated with mFOLFIRINOX. The x-axis of each plot represents CD107a expression, as indicated. All data are presented as the mean ± SD. Statistical significance was determined by *t*-test. *p* < 0.05 (*), *p* < 0.01 (**), *p* < 0.001 (***), *p* < 0.0001 (****). ns, no significant difference.

Our analysis revealed that mFOLFIRINOX treatment alone did not directly activate NK cells (Figure 3A and Figure S5); however, when NK cells were co-cultured with PDAC cell lines pre-treated with mFOLFIRINOX, we observed varied immune responses across the cell lines, with an overall enhancement in NK cell activation and cytotoxicity against the treated tumor targets, as shown in Figure 3.

For CFPAC-1 cells, identified as highly resistant to NK cells based on Real-Time Cell Analysis (RTCA) results (Figure 2C), mFOLFIRINOX pre-treatment resulted in the increased secretion of INF-γ and TNF-α (Figure 3A), as well as the elevated expression of INF-γ, TNF-α, CD107a, perforin, and granzyme B in NK cells (Figure 3B,C and Figure S6). These findings indicated the enhanced activation of NK cells against this cell line, previously characterized by significant resistance.

Conversely, the treatment of AsPC-1 cells, characterized by moderate resistance, led to the decreased secretion of INF-γ and TNF-α (Figure 3A). However, there was an increase in the expression of the activation markers CD107a, perforin, and granzyme B in NK cells (Figure 3B,C and Figure S7), indicating that mFOLFIRINOX pre-treatment could modulate the immune response.

In the case of PANC-1 cells, which are highly sensitive to NK cells, mFOLFIRINOX pre-treatment led to a decrease in INF-γ secretion; however, its levels remained high. There was no significant change in TNF-α secretion or the expression of other key markers (Figure 3 and Figure S8). This pattern suggests a nuanced immune response to mFOLFIRINOX pre-treatment, particularly in cell lines sensitive to NK cells.

These findings demonstrated that mFOLFIRINOX pre-treatment could selectively modulate the immune response of NK cells, significantly enhancing their activation and cytotoxic potential against PDAC cell lines. This is particularly notable in cases where resistance to NK cells was previously evident.

### 3.4. Assessment of NK-Cell-Activating Ligand and Apoptosis-Inducing Receptor Expression Following mFOLFIRINOX Treatment in a PDAC Animal Model

We further used an in vivo PDAC mouse model to evaluate the effects of mFOLFIRINOX treatment on the expression of NK-cell-activating ligands and apoptosis-inducing receptors using the AsPC-1 cell line. Preliminary tests showed that mFOLFIRINOX doses twice or five times the standard concentrations (oxaliplatin 1.5 mg/kg, irinotecan 2 mg/kg, 5-fluorouracil 4 mg/kg, leucovorin 2 mg/kg) led to severe toxicity in mice (Figures S9–S12). As a result, we opted for standard and reduced doses in our experiments.

**Figure 4:**
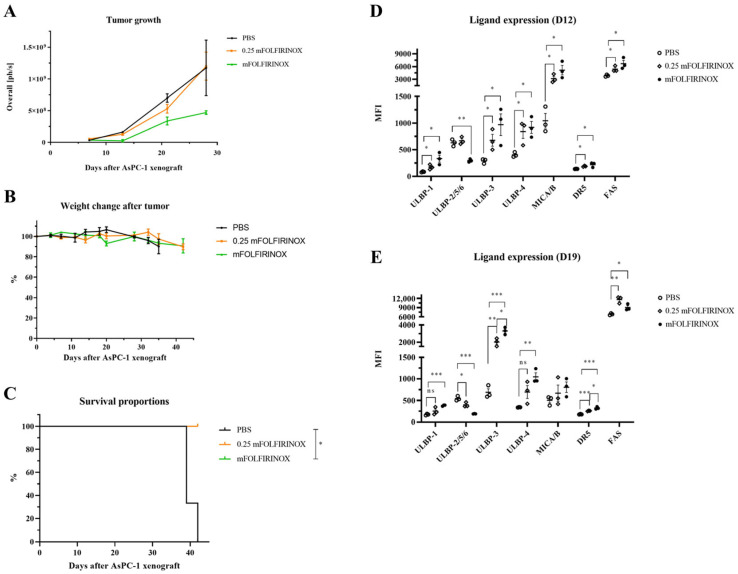
Evaluation of the effects of mFOLFIRINOX in a PDAC mouse model. Tumor burden was measured weekly post-implantation by bioluminescence imaging (BLI) and quantified as photons per second (ph/s) (**A**). Body weight changes were monitored twice weekly post-implantation (**B**). Kaplan–Meier survival curves were generated for AsPC-1/Luc-bearing mice (**C**). The expression levels of NK-cell-activating ligands and apoptosis-inducing receptors were evaluated by flow cytometry on days 12 and 19 post-implantation after harvesting AsPC-1/Luc cells from the peritoneal cavity (**D**,**E**). The groups evaluated were as follows: PBS-only (PBS, black), 25% standard dose mFOLFIRINOX (0.25 mFOLFIRINOX, orange), and standard dose mFOLFIRINOX (mFOLFIRINOX, green). All panels (**A**–**E**) were generated using n = 3 mice per group. Data for tumor burden, weight changes, and ligand expression are presented as the mean ± SEM. The statistical significance for ligand expression and survival was determined by *t*-test and log-rank test, respectively. *p* < 0.05 (*), *p* < 0.01 (**), *p* < 0.001 (***), ns, no significant difference.

The findings revealed that tumor growth was significantly inhibited by a standard dose of mFOLFIRINOX compared with a reduced dose, which was less effective (Figure 4A). Importantly, the mice tolerated the standard dose well, showing no significant changes in body weight with extended survival rates in the mFOLFIRINOX group compared with the PBS control group (Figure 4B,C).

Analysis of tumor cells extracted from the peritoneal cavity at 12 and 19 days post-tumor implantation revealed significant changes in ligand and receptor expression (Figure 4D,E and Figure S13). By day 12, the mFOLFIRINOX group exhibited a marked increase in the expression of the NKG2D ligands ULBP-1, ULBP-3, ULBP-4, and MIC A/B. A notable decrease in ULBP-2/5/6 expression, which was not evident in the reduced-dose group, was also observed. Furthermore, the expression levels of FAS and DR5 were significantly elevated. On day 19, these trends persisted, except for MIC A/B expression, which did not increase further (Figure 4D,E). Importantly, the expression levels of ULBP-1 and ULBP-4 remained higher in the mFOLFIRINOX group than in the PBS group, which was not observed in the reduced-dose group.

These results indicated that mFOLFIRINOX pre-treatment at a standard dose could significantly enhance NK cell cytotoxicity through the upregulation of activating ligands and apoptosis-inducing receptors in PDAC cells. The increase in tumor suppression efficacy, along with a tolerable toxicity profile, underscores the potential of this dosing strategy for further experiments.

### 3.5. Anti-Cancer Efficacy of mFOLFIRINOX and NK Cell Combination Therapy in a PDAC Animal Model

Using our PDAC animal model, we assessed the anti-cancer efficacy of combining mFOLFIRINOX with NK cell therapy. NK cells, harvested weekly from the PBMCs of a donor over three treatment cycles, underwent rigorous verification for purity, phenotype, and cytotoxicity using flow cytometry prior to administration in mice. The cytotoxic functionality of NK cells was validated through co-culture with K562 cells for 4 h at an effector-to-target cell (E:T) ratio of 4:1, which confirmed their functional integrity (Table 2 and Figure S14) [24].

**Figure 5:**
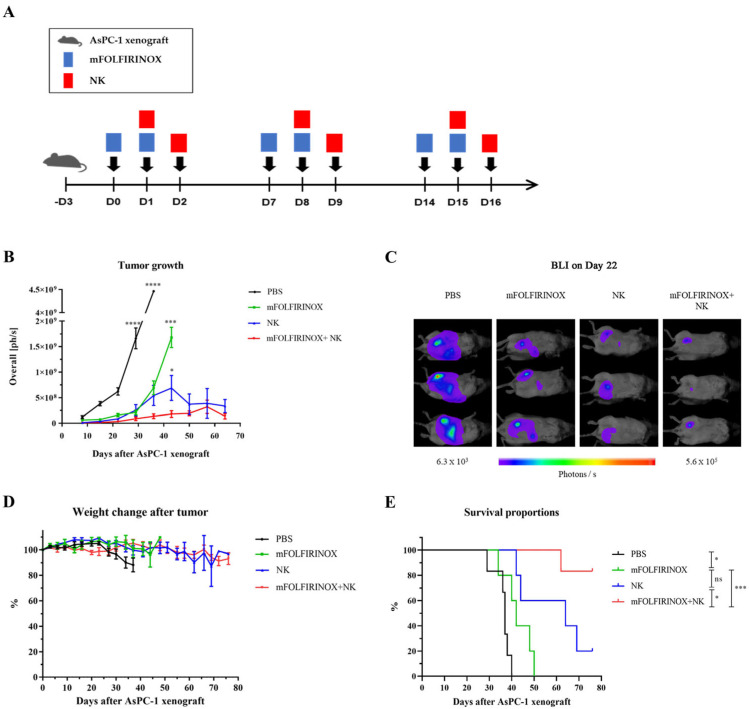
Evaluation of the anti-cancer efficacy of mFOLFIRINOX and NK cell combination therapy in a PDAC mouse model. The experimental design for in vivo treatment is illustrated, where NSG mice were intraperitoneally inoculated with 1 × 10^6^ AsPC-1/Luc cells on day −3 to establish the xenograft model, and treatments with mFOLFIRINOX (blue rectangles) and NK cells (red rectangles) were administered according to the schedule from day 0, repeated weekly for three cycles (**A**). Tumor burden was measured weekly post-implantation by bioluminescence imaging (BLI) and quantified as photons per second (ph/s), presented as the mean ± SEM. In panel (**B**), statistical significance was denoted for comparisons with the mFOLFIRINOX+NK group. Representative bioluminescence images from three mice in each group are shown (ventral dorsal view) on day 22 (**C**). Changes in body weight were monitored twice weekly post-implantation, and the data are presented as the mean ± SEM (**D**). The Kaplan–Meier survival of AsPC-1/Luc-bearing mice was assessed (**E**). The statistical significance for tumor burden and survival was determined by one-way ANOVA and log-rank test, respectively. *p* < 0.05 (*), *p* < 0.001 (***), *p* < 0.0001 (****). ns, no significant difference.

An intraperitoneal PDAC xenograft model was established, and mice were treated with mFOLFIRINOX, NK cells, or their combination according to the indicated schedule to assess therapeutic efficacy (Figure 5A). In this xenograft model, bioluminescence imaging revealed disseminated peritoneal tumor spread, as well as localized signals near the pancreatic region (Figure S15). In the PBS control group, rapid tumor growth and weight loss were observed, leading to mortality starting from day 29 (Figure 5B–E). Conversely, in the mFOLFIRINOX group, tumor growth was slower until day 19; however, it showed rapid progression after the treatment schedule concluded. BLI on day 22 detected significant peritoneal tumor dissemination in the PBS group, whereas the treated groups (mFOLFIRINOX, NK, and mFOLFIRINOX+NK) showed a marked reduction in tumor burden (Figure 5C). Specifically, the tumor burden was slightly larger in the mFOLFIRINOX group than in the NK and mFOLFIRINOX+NK groups. Notably, the mFOLFIRINOX+NK group exhibited significantly prolonged tumor suppression and an improvement in survival rates, outperforming all other groups (Figure 5B,E).

**Figure 6:**
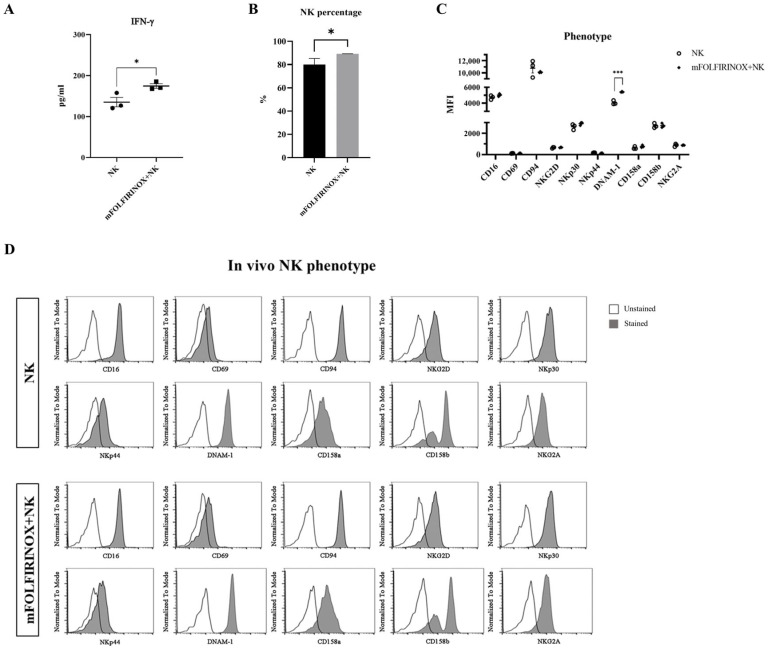
Evaluation of NK cell function and immune response in a PDAC mouse model. On day 24 post-tumor implantation, NK cells were harvested from the peritoneal cavity, and the serum was collected for the assessment of phenotype and INF-γ expression, respectively (n = 3/group). INF-γ concentration in the serum was measured by ELISA (**A**). The proportion of CD3^−^CD56^+^ NK cell population among live CD45^+^ cells was analyzed (**B**). The expression levels of various markers in live CD45^+^CD3^−^CD56^+^ cells were analyzed (**C**). Representative phenotype expression for a mouse in each group is presented as histogram (**D**). All data are presented as the mean ± SEM. Statistical significance was determined by *t*-test. *p* < 0.05 (*), *p* < 0.001 (***).

After the completion of all treatments on day 19, serum INF-γ levels were assessed on day 24, which showed a significant increase in the mFOLFIRINOX+NK group compared with the NK group (Figure 6A). In addition, analysis of peritoneal lavage cells demonstrated a significantly higher proportion of CD3^−^CD56^+^ NK cells in the mFOLFIRINOX+NK group compared to the NK group (Figure 6B and Figure S16). This elevation suggests that combination therapy could not only impede tumor growth and improve survival rates but also enhance NK cell activation and persistence, as demonstrated by the increased secretion of INF-γ and the higher proportion of NK cells in peritoneal cavity. Taken together, the findings underscore the superior efficacy of combination therapy in PDAC treatment, indicating its potential as an effective therapeutic strategy.

### 3.6. Analysis of Phenotypic Changes in NK Cells Following mFOLFIRINOX and NK Cell Combination Therapy in a PDAC Animal Model

The final phase of our investigation focused on the analysis of phenotypic changes in NK cells in a PDAC animal model following the administration of mFOLFIRINOX and NK cells. A comprehensive analysis was carried out on day 24 post-tumor implantation with all experimental groups.

Notably, there was a significant increase in serum IFN-γ levels in the mFOLFIRINOX+NK group compared with the NK group (Figure 6A). This elevation in IFN-γ suggests an enhanced anti-cancer immune response, highlighting the effectiveness of combination therapy in augmenting NK-cell-mediated anti-tumor activity. In addition, NK cell proportion (CD3^−^CD56^+^ population) was higher in the combination group compared to NK monotherapy (Figure 6B). With the exception of DNAM-1, NK cells exhibited no significant phenotypic differences across the experimental groups. This finding suggests that, for the most part, mFOLFIRINOX treatment supports NK cell persistence without impairing their functional phenotype (Figure 6B–D).

These findings demonstrated that mFOLFIRINOX and NK cell combination therapy could not only preserve NK cell persistence and function but also significantly enhance the immune response against PDACs. The maintenance of NK cell phenotypes, together with their higher proportion and a notable increase in IFN-γ secretion, underscores the synergistic efficacy of this therapeutic strategy in fighting pancreatic cancer.

## 4. Discussion

In PDAC, identifying effective and safe therapeutic strategies remains a significant challenge. Characterized by aggressive progression and resistance to conventional therapies, PDAC necessitates innovative treatment approaches [7,26]. Although the first-line chemotherapeutic regimen mFOLFIRINOX is recognized for its efficacy, its use is often limited by significant toxicity and modest response rates of less than 32% [6,7,20,22,27]. Therefore, novel therapeutic strategies are urgently needed not only to improve efficacy and toxicity profiles but also to disrupt the pathological tumor ecosystem through coordinated cytotoxic and microenvironment-modulating actions [28].

NK cells, key components of the body’s initial barrier against viral infections and tumors, play a vital role in the immune system without prior sensitization [29,30]. From an ecological perspective that views cancer as interacting tumor subpopulations within dynamic niches [28], NK cells restrain heterogeneous clones through antigen-agnostic recognition of stress ligands and missing-self surveillance. In contrast, T cells require peptide-MHC presentation and therefore primarily target antigen-defined clones. In parallel, NK-derived cytokines and chemokines recruit and mature intratumoral cDC1 and prime CD8^+^ T-cell responses, collectively remodeling the tumor microenvironment [31]. Notably, compared with T cell therapies, NK cell therapy has a lower risk of cytokine release syndrome (CRS) or graft-versus-host disease (GvHD), making it an attractive therapeutic approach [7,30].

Clinical experience with NK therapy shows activity with generally favorable safety, though resistance often limits durability. In hematologic malignancies, adoptive infusion of haploidentical NK cells in refractory AML achieved complete remission in a subset after high-dose cyclophosphamide/fludarabine lymphodepletion (5/19 CRs) [32]. Cord-blood-derived CD19 CAR-NK cells induced responses in 73% of heavily pre-treated B-cell malignancies without CRS or ICANS [33]. Pre-complexing cytokine-activated cord-blood NK cells with the CD30xCD16A engager AFM13 yielded an overall response rate 92.9% with reassuring tolerability in CD30-positive lymphoma [34]. In solid tumors, NK-modulating strategies have shown encouraging but less consistent activity compared to hematologic malignancies. For example, the EGFRxCD16A engager AFM24 demonstrated acceptable safety/pharmacodynamics with disease stabilization and occasional partial responses, and ex vivo expanded NK cells (SNK01) improved ORR and PFS when added to pembrolizumab in a small randomized NSCLC study [35,36]. Feasibility of local or regional delivery has also been shown (intracranial HER2 CAR-NK-92 in recurrent glioblastoma; hepatic–arterial infusion of high-dose autologous NK cells following hepatic arterial infusion chemotherapy in advanced hepatocellular carcinoma) with manageable safety and early activity [23,37]. Nonetheless, tumor-intrinsic and microenvironmental mechanisms, including TGF-β/adenosine-driven suppression, hypoxia and metabolic stress, protease-mediated shedding or down-modulation of activating ligands (e.g., MICA/B) that blunt NKG2D signaling, upregulation of HLA-E engaging the inhibitory NKG2A receptor, and ADAM17-dependent cleavage of CD16 that dampens ADCC can attenuate responses and shorten benefit windows, motivating combinations and engineering to enhance persistence, trafficking, and resistance to checkpoint-mediated suppression [10,38,39,40].

PDAC is known for its dense desmoplastic stroma and hypoxic TME, comprising various immunosuppressive cells such as pancreatic stellate cells (PSCs), myeloid-derived suppressor cells (MDSCs), tumor-associated macrophages (TAMs), and regulatory T cells (Tregs) [29,41]. This ‘cold’ TME results in an overall response rate of only 0–3% to immune checkpoint blockade (ICB) therapies [41]. Retrospective studies in PDAC patients have demonstrated that selected patients treated with combination ICB plus chemotherapy have considerably improved median overall survival (OS) by as much as 12 months compared to chemotherapy alone [42]. Together, these observations underscore the need to approach PDAC as a pathological ecosystem, pursuing multifaceted strategies that simultaneously reduce tumor burden and reprogram the stromal/immune niches.

In PDAC, the dysfunction of NK cells, characterized by disrupted infiltration and impairment of cytotoxic degranulation, contributes to the development and progression of the disease [29,30,43,44]. Despite these constraints, PDAC-directed NK therapies are progressing in preclinical and early clinical settings [7,18,29,30,45]. Recent PDAC reports of NK cell therapy (primarily case-level) show feasibility with signals of activity, and a multimodal regimen pairing chemotherapy with the IL-15 superagonist N-803 and PD-L1-targeted high-affinity NK has shown survival signals in heavily pre-treated disease [46,47]. Preclinically, the STING agonist cGAMP both directly activates NK cells and sensitizes PDAC cells (e.g., ULBP2/5/6 upregulation and apoptosis), and, in combination with mesothelin-targeted CAR-NK-92, achieves superior tumor control and prolongs survival in mouse models [45]. Taken together with these emerging clinical and preclinical signals, the decreased expression of MHC class I in PDAC progression further supports NK cell therapy in this context [41,43].

Each component of the mFOLFIRINOX regimen has been shown to increase NK-cell-activating ligand expression in tumor cells. Additionally, 5-fluorouracil selectively kills MDSCs, thereby enhancing T-cell-mediated anti-tumor immunity [29,41,42]. Clinical observations also support this immunomodulatory effect: Van Eijck et al. (2024) reported that mFOLFIRINOX increased circulating levels of IL-15 and IL-18, cytokines that stimulate the proliferation of both NK and T cells [48], while Zwart et al. (2023) demonstrated a reduction in Tregs and CD163^+^ macrophages, alongside increased frequencies of cytotoxic T cells and CD163^−^ macrophages in patients receiving this regimen [49]. Considering the individual benefits of mFOLFIRINOX and NK cell therapy, their combination may have a synergistic effect. However, no study has investigated this combined therapeutic approach, which prompted us to investigate the efficacy of this novel therapeutic strategy in the treatment of PDAC.

In our study, we investigated the synergistic effect of this approach in vitro and in vivo in a PDAC xenograft model. We observed that chemotherapy, particularly mFOLFIRINOX, could not only directly kill tumor cells but also modulate NK cell ligand expression on tumor cells, thereby enhancing NK cell activation. Previous studies, such as the study conducted by Koh et al. (2023) [18], have illustrated how chemotherapeutic agents such as gemcitabine can augment NK cell activity. This augmentation occurs through the increased expression of NKG2D ligands on cancer cells, making them more susceptible to NK-cell-mediated cytotoxicity [18].

The chemotherapeutic agents comprising mFOLFIRINOX—oxaliplatin, irinotecan, and 5-fluorouracil—have been shown to enhance NK-cell-mediated cytotoxicity by upregulating ligands such as NKG2D, DNAM-1, and NKp30 ligands across various tumor types, including pancreatic cancer [11,12,13,14,15,19]. These effects increase tumor cell susceptibility to NK cells and complement their natural cytotoxic function, which relies on a dynamic balance of activating and inhibitory signals [10]. Our findings align with prior studies reporting that, as part of their immunomodulatory effects, standard chemotherapeutic agents can upregulate NK-cell-activating ligands and death receptors, thereby enhancing tumor susceptibility to NK cell recognition and killing. In our experimental system, mFOLFIRINOX treatment alone did not directly activate NK cells (Figure 3A and Figure S5); rather, pre-treatment of PDAC tumor cells with mFOLFIRINOX enhanced NK cell activation and cytotoxicity upon subsequent co-culture, underscoring the tumor-intrinsic immunogenic modulation driving this effect. Consistent with previous reports, we observed increased expression of NK-cell-activating ligands and apoptosis-inducing receptors in PDAC cells following mFOLFIRINOX treatment (Figure 1). This upregulation led to increased NK cell activation and cytotoxicity (Figure 2), as evidenced by the upregulation of CD107a, perforin, and granzyme B (Figure 3).

A notable limitation of our study is the observed variation in the NK cell immune response across different PDAC cell lines when subjected to combination therapy despite the therapy demonstrating superior cytotoxicity overall (Figure 2 and Figure 3). For example, CFPAC-1 cells—initially resistant to NK killing (Figure 2C and Figure S3E)—showed marked increases in cytokine secretion and cytotoxic marker expression following mFOLFIRINOX pre-treatment (Figure 2C, Figure 3 and Figure S6). In contrast, PANC-1 cells, which were already sensitive to NK cells, did not show further enhancement with combination therapy (Figure 2A, Figure 3 and Figure S8).

These distinct responses may, at least in part, be influenced by intrinsic tumor differentiation status. Previous in vitro studies, although conducted in colon cancer cell lines, have reported that poorly differentiated tumor cells were more susceptible to NK cell cytotoxicity than well-differentiated ones [14]. This observation parallels our findings (Figure 2), where PANC-1 cells (poorly differentiated [50]) exhibited high baseline NK sensitivity, AsPC-1 cells (moderately to highly differentiated [51,52]) showed intermediate sensitivity, and CFPAC-1 cells (well-differentiated [51]) demonstrated low NK sensitivity in our assays.

NK cells, when activated against abnormal target cells, exert dual effects by releasing cytotoxic granules (e.g., perforin and granzyme B) to induce direct cell killing and by secreting cytokines for immune regulation. The release of cytotoxic granules and cytokines by NK cells is regulated by distinct mechanisms [53,54]. Upon activation by target cells, NK cells form an immune synapse (IS) through which cytotoxic granules are efficiently secreted, and cytokines such as IFN-γ and TNF-α are released across the entire cell surface [53]. The killing process by NK cells is a highly regulated sequence, effectively inducing target cell apoptosis through IS-mediated granule release, followed by detachment for serial killing [55,56,57,58]. In cases of ineffective cell lysis, sustained IS and delayed detachment occur, resulting in sustained Ca^2+^ flux in NK cells. This activates transcription factors such as NFAT, CREB, and NFκB, promoting the hypersecretion of cytokines including IFN-γ and TNF-α [55,57].

In PANC-1 cells, which exhibited high baseline NK sensitivity with over 70% of NK cells expressing cytotoxic markers even before mFOLFIRINOX pre-treatment (Figure 3B,C), only a modest, non-significant further increase in cytotoxic molecule expression was observed after pre-treatment (Figure S8). IFN-γ secretion decreased, but the magnitude of reduction was less pronounced than in AsPC-1 cells (Figure 3A), suggesting that, relative to AsPC-1, additional serial killing was likely limited due to already efficient NK–target interactions.

Interestingly, in AsPC-1 cells, we observed enhanced degranulation and cytolysis in the mFOLFIRINOX+NK group compared to NK monotherapy, yet cytokine secretion was reduced (Figure 2B and Figure 3). This suggests efficient serial killing and rapid detachment due to heightened target susceptibility. In contrast, for CFPAC-1 cells, which were not inherently susceptible to NK cell monotherapy (Figure 2C), combination therapy induced both strong cytotoxic and cytokine responses (Figure 3)—highlighting the dynamic interplay between chemotherapy-induced immunogenic modulation and baseline NK sensitivity. Although combination therapy is promising, further investigation of the molecular and cellular mechanisms underlying cell-line-specific variability is required. A better understanding of these mechanisms could pave the way for the development of more tailored and effective treatment strategies for PDAC patients, ensuring that therapy is appropriate according to each individual’s tumor biology.

Our study required an animal model that could recapitulate key aspects of PDAC progression and relevant clinical features observed in patients. Our study showed that intraperitoneal implantation results in widespread peritoneal dissemination (Figure 5C), with tumor accumulation near the pancreas (Figure S15), along with malignant ascites and cachexia—hallmarks of advanced-stage PDAC [59,60,61,62,63,64,65]. By establishing a peritoneal carcinomatosis-like dissemination pattern that mimics advanced-stage PDAC, we demonstrated the anti-cancer efficacy of our combination therapy.

In preliminary dose-finding studies, high concentrations of mFOLFIRINOX resulted in the occurrence of melena, indicative of upper gastrointestinal bleeding, and led to early mortality due to severe toxicity rather than tumor progression (Figures S9–S12). Due to these adverse effects, we experimented with reduced doses of mFOLFIRINOX, specifically one-fifth and one-twentieth of the original concentration, to achieve an optimal balance between tolerability, tumor growth inhibition, and the expression of NK-cell-activating ligands (Figure 4). In contrast to higher concentrations, neither the one-fifth dose nor the one-twentieth dose (0.25 mFOLFIRINOX group) induced severe toxicity in mice and or adversely affected body weight or survival (Figure 4B,C). Although the 0.25 mFOLFIRINOX group was not considerably different from the PBS group, the mFOLFIRINOX group demonstrated delayed tumor growth (Figure 4A). In agreement with our in vitro results, mFOLFIRINOX pre-treatment was found to increase the expression of NKG2D ligands and apoptosis-inducing receptors, with a greater increase in NK-cell-activating ligand expression in the mFOLFIRINOX group compared with the 0.25 mFOLFIRINOX group (Figure 4D,E). Consequently, this led to the selection of the mFOLFIRINOX concentration that was tolerable and effective in inhibiting tumor growth and enhancing NK-cell-activating ligand expression for subsequent in vivo experiments.

In our mouse xenograft model, the mFOLFIRINOX+NK group exhibited significantly prolonged tumor suppression and improved survival rates, outperforming all other treatment groups (Figure 5B–E). The enhanced tumor eradication observed with combination therapy may be attributed not only to the direct killing effect of mFOLFIRINOX but also to the increased susceptibility of tumor cells to NK cell cytotoxicity. This increase in susceptibility led to an enhanced immune response in vivo, as evidenced by more effective tumor cell destruction and IFN-γ secretion (Figure 5 and Figure 6A). Furthermore, the NK cell population was maintained at higher levels in the combination group compared to NK monotherapy, and DNAM-1 expression was elevated (Figure 6B–D), while other phenotypic markers remained largely unchanged. The preservation of NK cell function with higher NK proportion following mFOLFIRINOX treatment (Figure 6B–D) suggests the feasibility of this combination therapy in clinical settings. These phenotypic findings will be further investigated in ongoing clinical studies evaluating ex vivo expanded human NK cells combined with first-line mFOLFIRINOX chemotherapy in patients with advanced PDAC to more comprehensively characterize NK cell dynamics in patients.

Although the NSG mouse model can offer valuable insights into the progression of PDAC and the efficacy of our combination therapy, it falls short of fully capturing the complexities of the human immune system. This limitation is particularly relevant when considering the potential immune response in patients following NK cell therapy. The inability of our model to replicate the intricate and reactive nature of the human immune environment prevents a complete understanding of how our therapy might perform under actual conditions. Therefore, a cautious interpretation of our findings and further validation in more immunocompetent models or human clinical trials are required, which are essential to accurately establish the efficacy and safety of our combination therapy in clinical settings.

RTCA-based cytotoxicity assays demonstrated that combining NK cells with a 10-fold reduced dose of mFOLFIRINOX achieved substantial antitumor effects, comparable to those observed with a higher mFOLFIRINOX dose alone (Figure S2E). This synergy suggests that our combination approach could reduce systemic toxicity by lowering chemotherapy requirements without compromising efficacy. Supporting this, in vivo experiments confirmed that the selected mFOLFIRINOX dose was both well tolerated and effective when paired with NK cell therapy. Together with clinical data supporting NK cell safety [23], these findings highlight the translational potential of this strategy in achieving a favorable balance of efficacy and safety in PDAC treatment.

In this study, we utilized blood samples from healthy donors to investigate the anti-cancer efficacy of combination therapy with ex vivo expanded NK cells. Despite impaired NK cell function in PDAC patients, a study by Lim et al. (2019) demonstrated that ex vivo expanded NK cells could regain functionality and induce the destruction of PDAC cell lines [43]. Although these findings are promising, it should be noted that variations in the feeder cells and cytokines used for expansion could influence outcomes. Consequently, further research using ex vivo expanded NK cells derived from PDAC patients is essential to confirm the potential of our therapeutic approach, which is currently being evaluated in ongoing clinical trials in our group.

Our study presents a novel and promising strategy for treating advanced PDAC. By synergistically combining mFOLFIRINOX with NK cell therapy, our approach could not only directly target tumor cells but also modulate ligands that activate NK cells, significantly enhancing the therapeutic potential. This research paves the way for future clinical trials with the goal of improving patient outcomes for this difficult-to-treat disease. Furthermore, this study highlights the importance of continued investigation of the interactions between tumors and the immune system in PDAC, suggesting that a deeper understanding of these dynamics could lead to even more effective treatments.

## 5. Conclusions

In conclusion, our study introduces a promising therapeutic strategy for treating advanced PDAC by synergistically combining mFOLFIRINOX with NK cell therapy. This approach directly enhances the susceptibility of PDAC cells to NK-cell-mediated cytotoxicity through NK-cell-activating ligands and apoptosis-inducing receptors. Our findings demonstrated that this combination therapy significantly inhibited tumor growth and prolonged survival in preclinical models, paving the way for future clinical trials aimed at improving outcomes for this challenging disease. Furthermore, it highlights the importance of continued investigation into the interactions between tumors and the immune responses in PDAC, suggesting that a deeper understanding of these dynamics could drive the development of even more effective treatments.

## Figures and Tables

**Table 1:** List of antibodies used in flow cytometry.

Target Antigen	Fluorochrome	Clone	Company	Catalog
Live/Dead (Ghost dye)	Ghost dye 510	-	Tonbo Biosciences (San Diego, CA, USA)	13-0870-T100
Human ULBP-1	PerCP	170818	R&D systems (Minneapolis, MN, USA)	FAB1380C
Human ULBP-2/5/6	Brilliant Violet (BV) 421	165903	BD bioscience (San Jose, CA, USA	748128
Human ULBP-3	Alexa Fluor (AF) 488	166510	R&D systems (Minneapolis, MN, USA)	FAB1517G-100UG
Human ULBP-4	APC	709116	R&D systems (Minneapolis, MN, USA)	FAB6285A
Human MICA/B	PE-Cy7	6D4	Thermo Fisher Scientific (Waltham, MA, USA)	MA5-38728
Human B7-H6	PE	JAM1EW	Thermo Fisher Scientific (Waltham, MA, USA)	12-6526-42
Human DR4 (CD261)	PE	DJR1	Biolegend (San Diego, CA, USA)	307206
Human DR5 (CD262)	FITC	DR-5-01-1	Thermo Fisher Scientific (Waltham, MA, USA)	A15750
Human FAS	AF 700	DX2	Biolegend (San Diego, CA, USA)	305648
Human CD112	APC	R2.477	Thermo Fisher Scientific (Waltham, MA, USA)	17-1128-42
Human CD155	BV 786	SKII.4	BD bioscience (San Jose, CA, USA	748273
Human CD3	PerCP	UCHT1	Biolegend (San Diego, CA, USA)	300428
Human CD56	AF700	TULY56	Thermo Fisher Scientific (Waltham, MA, USA)	56-0566-42
Human CD45	Pacific blue	HI30	Biolegend (San Diego, CA, USA)	304029
Human CD16	SB 600	eBioCB16 (CB16)	Thermo Fisher Scientific (Waltham, MA, USA)	63-0168-42
Human CD69	PE	FN50	Miltenyi bio (Bergisch Gladbach, Germany)	130-113-524
Human CD94	APC	HP-3D9	BD bioscience (San Jose, CA, USA	559876
Human NKp30	AF647	P30-15	BD bioscience (San Jose, CA, USA	558408
Human NKp44	PE	P44-8	BD bioscience (San Jose, CA, USA	558563
Human NKp46	PE-Cy7	9E2/NKp46	BD bioscience (San Jose, CA, USA	562101
Human NKG2D	BV 786	1D11	BD bioscience (San Jose, CA, USA	743560
Human DNAM-1	PE	DX11	BD bioscience (San Jose, CA, USA	559789
Human NKG2A	PE	131411	R&D systems (Minneapolis, MN, USA)	FAB1059P-100
Human CD158a	APC	HP-3E4	BD bioscience (San Jose, CA, USA	564319
Human CD158b	BV 786	CH-L	BD bioscience (San Jose, CA, USA	743455
Human CD107a	AF 647	H4A3	Biolegend (San Diego, CA, USA)	328612
Human IFN-r	PE	4S.B3	Thermo Fisher Scientific (Waltham, MA, USA)	12-7319-42
Human TNF-a	BV 650	MAb11	Thermo Fisher Scientific (Waltham, MA, USA)	416-7349-42
Human Granzyme B	PE	GB11	BD bioscience (San Jose, CA, USA	561142
Human Perforin	BV711	Dg9	Biolegend (San Diego, CA, USA)	308130

**Table 2:** Purity (CD3^−^CD56^+^), phenotype, and cytotoxicity of the established NK cell products prepared for in vivo injection.

	Purity (%)	CD16 (%)	CD69 (%)	CD94 (%)	NKG2D (%)	NKp30 (%)	NKp44 (%)	NKp46 (%)	Cytotoxicity (%)
1st Product	93.7	97.1	99.7	99.4	99.7	99.9	97.6	40.8	77.6
2nd Product	83.2	97.5	94.8	99.5	77	99.8	90.6	40.6	76.1
3rd Product	91.1	97.8	78.2	99.8	97.3	99.9	96.6	40.7	81.2

## Data Availability

The original contributions presented in this study are included in this article/Supplementary Materials; further inquiries can be directed to the corresponding authors.

## Supplementary Materials

The following supporting information can be downloaded at: https://www.mdpi.com/article/10.3390/cancers17172785/s1, Figure S1: Gating strategy for the analysis of NK-cell-activating ligands and apoptosis-inducing receptors on tumor cells; Figure S2: Dose optimization and cytotoxicity assessment of mFOLFIRINOX and NK cell combination therapy in AsPC-1 cells using RTCA; Figure S3: Real-time analysis of NK cell cytotoxicity against PDAC cell lines using RTCA; Figure S4: Gating strategy for evaluating NK cell functional responses after co-culture with mFOLFIRINOX-pre-treated pancreatic cancer cells; Figure S5: Assessment of NK cell activation in response to direct mFOLFIRINOX treatment; Figure S6: Raw flow cytometry data corresponding to the functional response of NK cells co-cultured with CFPAC-1 pancreatic cancer cells; Figure S7: Raw flow cytometry data corresponding to the functional response of NK cells co-cultured with AsPC-1 pancreatic cancer cells; Figure S8: Raw flow cytometry data corresponding to the functional response of NK cells co-cultured with PANC-1 pancreatic cancer cells; Figure S9: Schematic diagram of the dose selection process for mFOLFIRINOX in NSG mouse models [66,67,68,69]; Figure S10: Evaluation of tolerability and survival following mFOLFIRINOX treatment at different dose levels in a PDAC xenograft mouse model; Figure S11: Evaluation of the effects of mFOLFIRINOX in a PDAC mouse model; Figure S12: Evaluation of the effects of dose-reduced mFOLFIRINOX in a PDAC mouse model; Figure S13: Flow cytometric gating strategy for analysis of peritoneal cells following AsPC-1/Luc xenograft and mFOLFIRINOX treatment in vivo; Figure S14: Flow cytometry gating strategy for NK cell cytotoxicity assessment against K562 target cells (related to Table 2); Figure S15: Representative bioluminescence imaging (BLI) of tumor progression in the PDAC xenograft model following the experimental design presented in Figure 5; Figure S16: Gating strategy and purity analysis of NK cells harvested from the peritoneal cavity in the PDAC xenograft model.

## Abbreviations

The following abbreviations are used in this manuscript:

PDAC	Pancreatic ductal adenocarcinoma
mFOLFIRINOX	Modified FOLFIRINOX
NKs	Natural killer cells
ACT	Adoptive cell transfer
CAR	Chimeric antigen receptor
PBMCs	Peripheral blood mononuclear cells
CI	Cell index
TME	Tumor microenvironment
CRS	Cytokine release syndrome
GvHD	Graft-versus-host disease
PSCs	Pancreatic stellate cells
MDSCs	Myeloid-derived suppressor cells
TAMs	Tumor-associated macrophages
Tregs	Regulatory T cells
ICB	Immune checkpoint blockade
OS	Overall survival
IS	Immune synapse
