# Supplementary material for: Synergistic Anti-Tumor Efficacy of Modified FOLFIRINOX and NK Cell Therapy in Pancreatic Ductal Adenocarcinoma
Source: Cancers (Basel). 2025 Aug 26;17(17):2785. doi: 10.3390/cancers17172785 (PMC12427234; doi:10.3390/cancers17172785)
Supplement: Supplementary file 1 [file cancers-17-02785-s001.zip › cancers-3756035-supplementary.pdf]

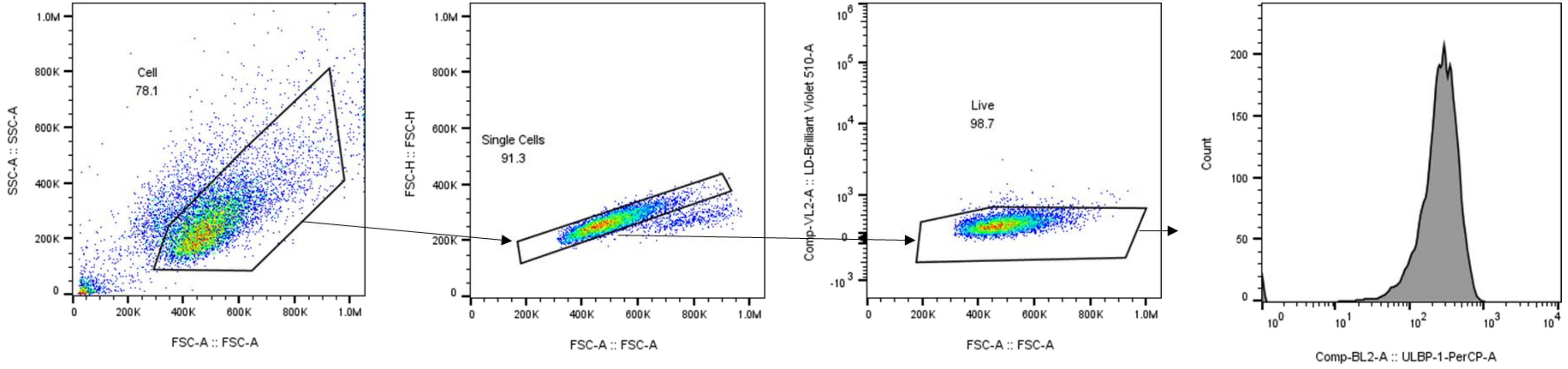


**Supplementary Figure S1.** Gating strategy for the analysis of NK cell-activating ligands and apoptosis-inducing receptors on tumor cells. Representative flow cytometry gating using AsPC-1 cells: initial gating was performed on FSC-A vs. SSC-A to select the cell population, followed by singlet gating using FSC-H vs. FSC-A. Live cells were identified by excluding Ghost Dye-positive dead cells. The expression of NK cell-activating ligands (ULBP-1, ULBP-2/5/6, ULBP-3, ULBP-4, MIC A/B, CD112, CD155, B7-H6) and apoptosis-inducing receptors (DR4, DR5, FAS) was subsequently analyzed within the gated live cell population.

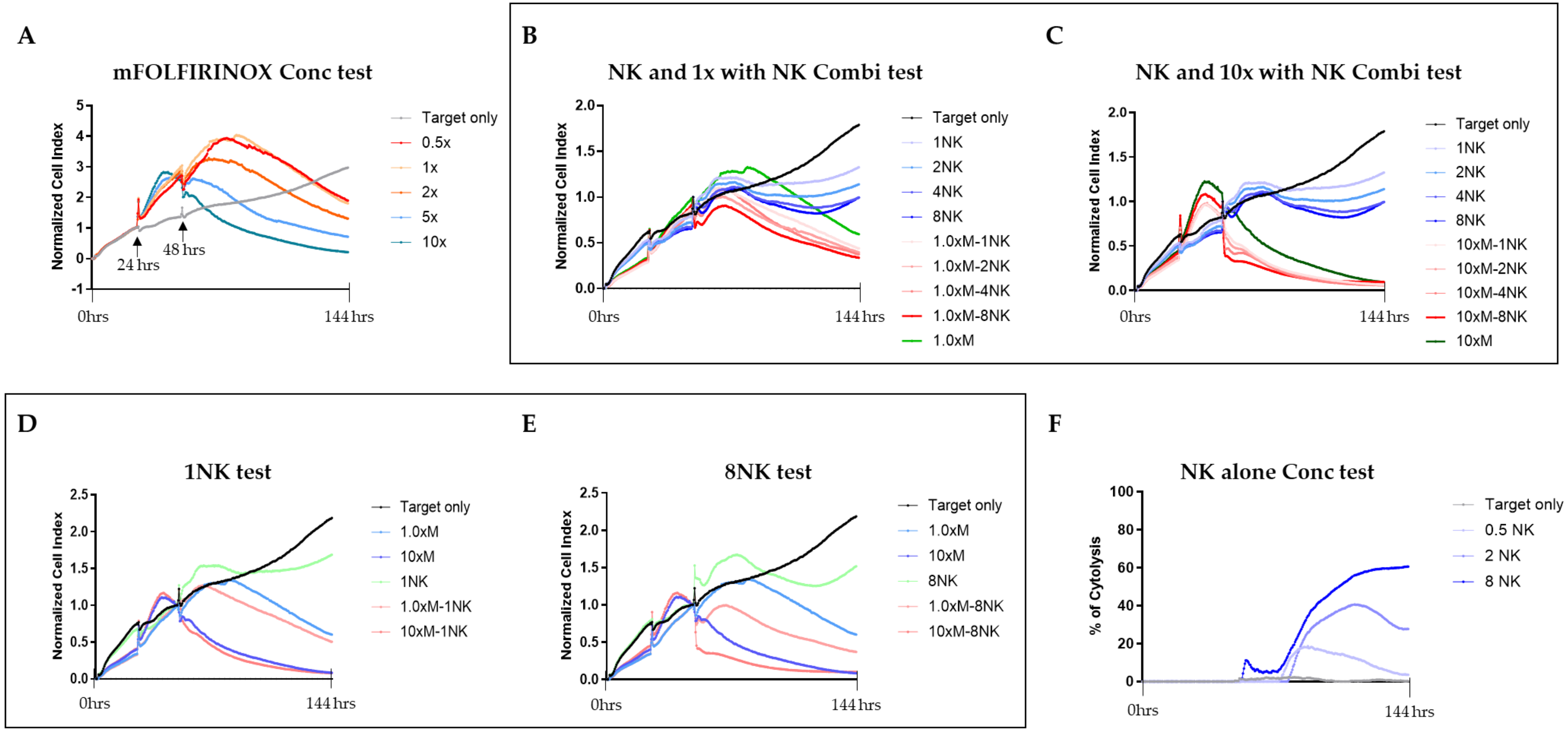


**Supplementary Figure S2.** Dose optimization and cytotoxicity assessment of mFOLFIRINOX and NK cell combination therapy in AsPC-1 cells using RTCA. AsPC-1 cells (Target cell only) were seeded at 1 × $10^4$ cells/well (time 0 h), and cell proliferation and cytotoxicity were monitored for a total of 144 h using Real-Time Cell Analysis (RTCA). mFOLFIRINOX was administered at 24 h post-seeding, and NK cells were added at 48 h post-seeding at the indicated effector-to-target (E:T) ratios. (A) AsPC-1 cells were treated with escalating doses of mFOLFIRINOX (0.5×, 1×, 2×, 5×, 10×; where 1× corresponds to oxaliplatin 2.14 µM, irinotecan 2.56 µM, 5-fluorouracil 92.2 µM, and leucovorin 8.45 µM).(B) mFOLFIRINOX (1.0×M), NK cells alone (1NK, 2NK, 4NK, 8NK), and their combinations were evaluated.(C) mFOLFIRINOX (10×M), NK cells alone (1NK, 2NK, 4NK, 8NK), and their combinations were evaluated.(D) Comparative evaluation of mFOLFIRINOX (1.0×M and 10×M), NK cells (1NK), and combinations (1.0×M-1NK and 10×M-1NK).(E) Comparison of high-dose NK cells (8NK) combined with mFOLFIRINOX (1.0×M and 10×M).(F) Dose-response of NK cells alone (0.5NK, 2NK, 8NK).

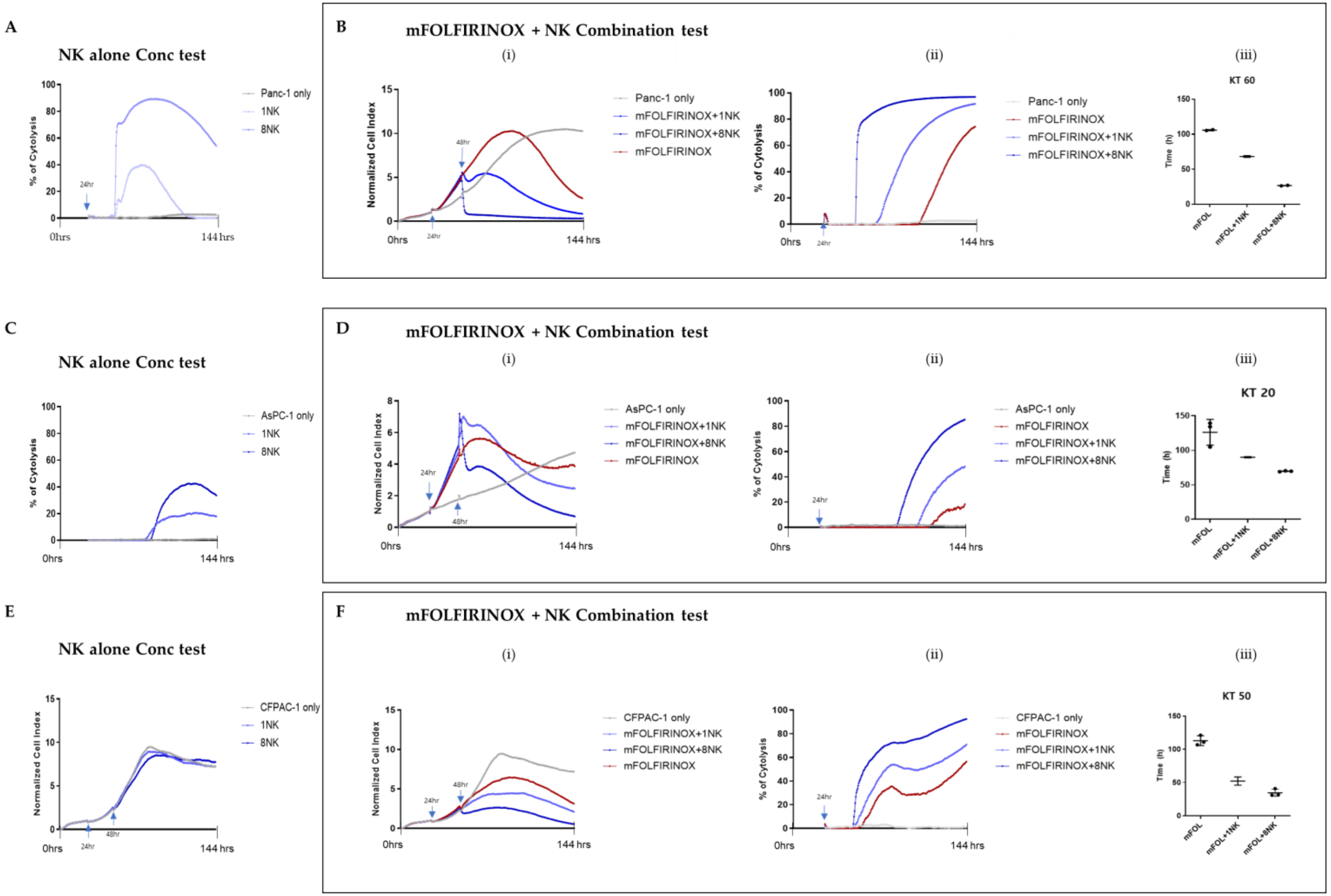


**Supplementary Figure S3.** Real-time analysis of NK cell cytotoxicity against PDAC cell lines using RTCA. RTCA was performed on PANC-1 (A, B), AsPC-1 (C, D), and CFPAC-1 (E, F) cells.(A, C, E) NK cell-mediated cytotoxicity was evaluated at E:T ratios of 1:1 (1NK) and 8:1 (8NK), with untreated target cells as controls (Target only). Effects of mFOLFIRINOX combined with NK cells on PDAC cells were assessed. The groups included Target only, mFOLFIRINOX alone, mFOLFIRINOX+1NK, and mFOLFIRINOX+8NK. mFOLFIRINOX was applied at the following concentrations: oxaliplatin 2.14 μM, irinotecan 2.56 μM, 5-fluorouracil 92.2 μM, and leucovorin 8.45 μM. Each panel displays: (i) Normalized cell index, (ii) Cytolysis (%), and (iii) Killing Time (KT), which indicates the time required to achieve a defined level of cytolysis. KT values were calculated as KT60 for PANC-1 (B), KT20 for AsPC-1 (D), and KT50 for CFPAC-1 (F), reflecting the time required to reach 60%, 20%, and 50% cytolysis, respectively.

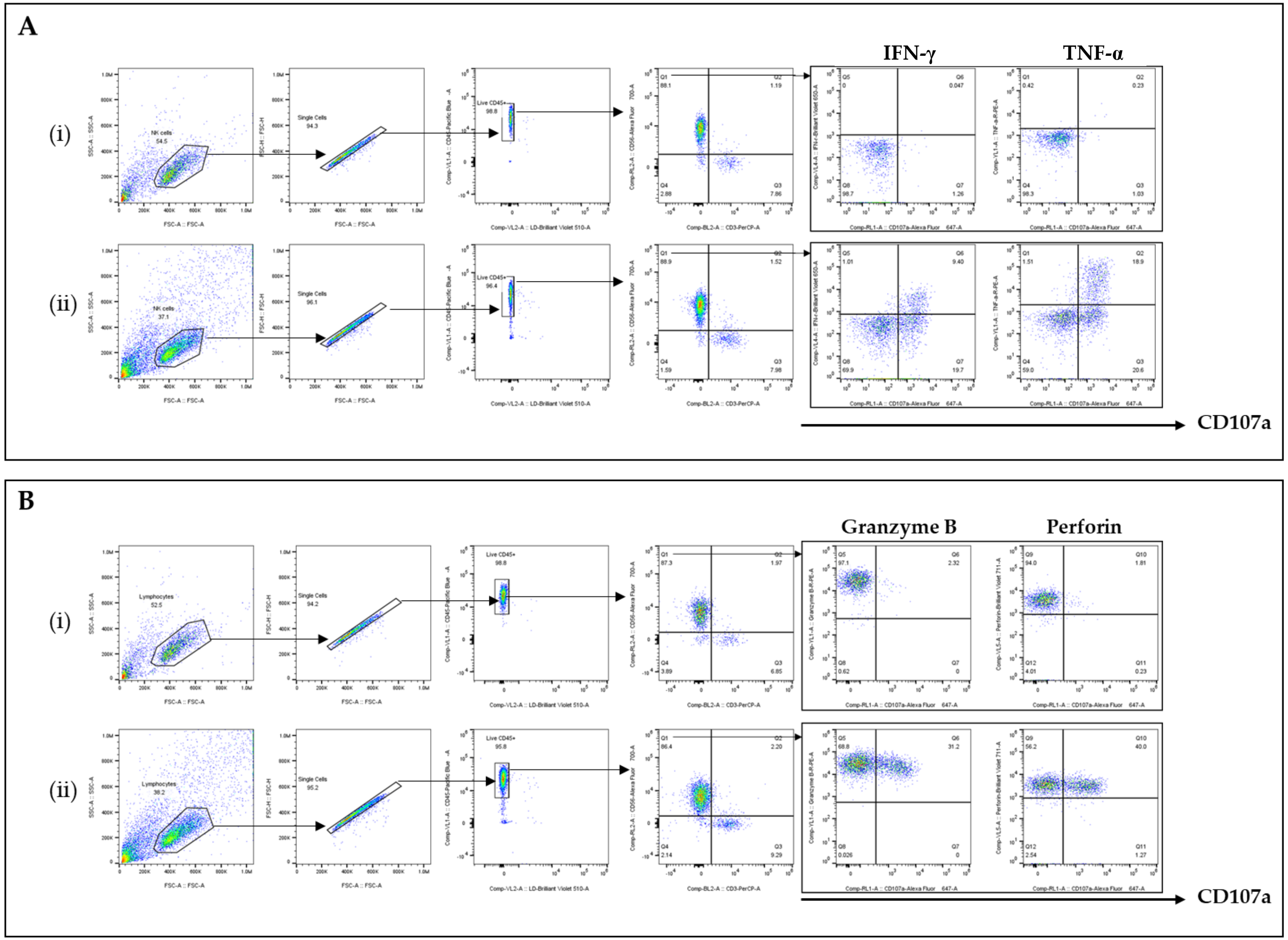


**Supplementary Figure S4.** Gating strategy for evaluating NK cell functional responses after co-culture with mFOLFIRINOX-pretreated pancreatic cancer cells. To assess NK cell function, NK cells were co-cultured with CFPAC-1 cells pre-treated with mFOLFIRINOX at effector-to-target (E:T) ratios of 2:1. cells, displayed as CD107a (x-axis) versus cytokines (y-axis). Subpanels: (i) NK cells only; (ii) NK cells co-cultured with CFPAC-1 cells.(Initial gating was performed on FSC-A vs SSC-A to identify target populations, followed by singlet gating (FSC-A vs FSC-H), live $CD45^+$ cell gating, and subsequent CD3 vs CD56 gating to identify NK cells ($CD45^+CD3^-CD56^+$).(A) Cytokine production (IFN-γ and TNF-α) was analyzed within $CD3^-CD56^+$ NK B) Cytotoxic molecule expression (granzyme B and perforin) was analyzed within $CD3^-CD56^+$ NK cells, displayed as CD107a (x-axis) versus cytotoxic molecules (y-axis). Subpanels: (i) NK cells only; (ii) NK cells co-cultured with CFPAC-1 cells.

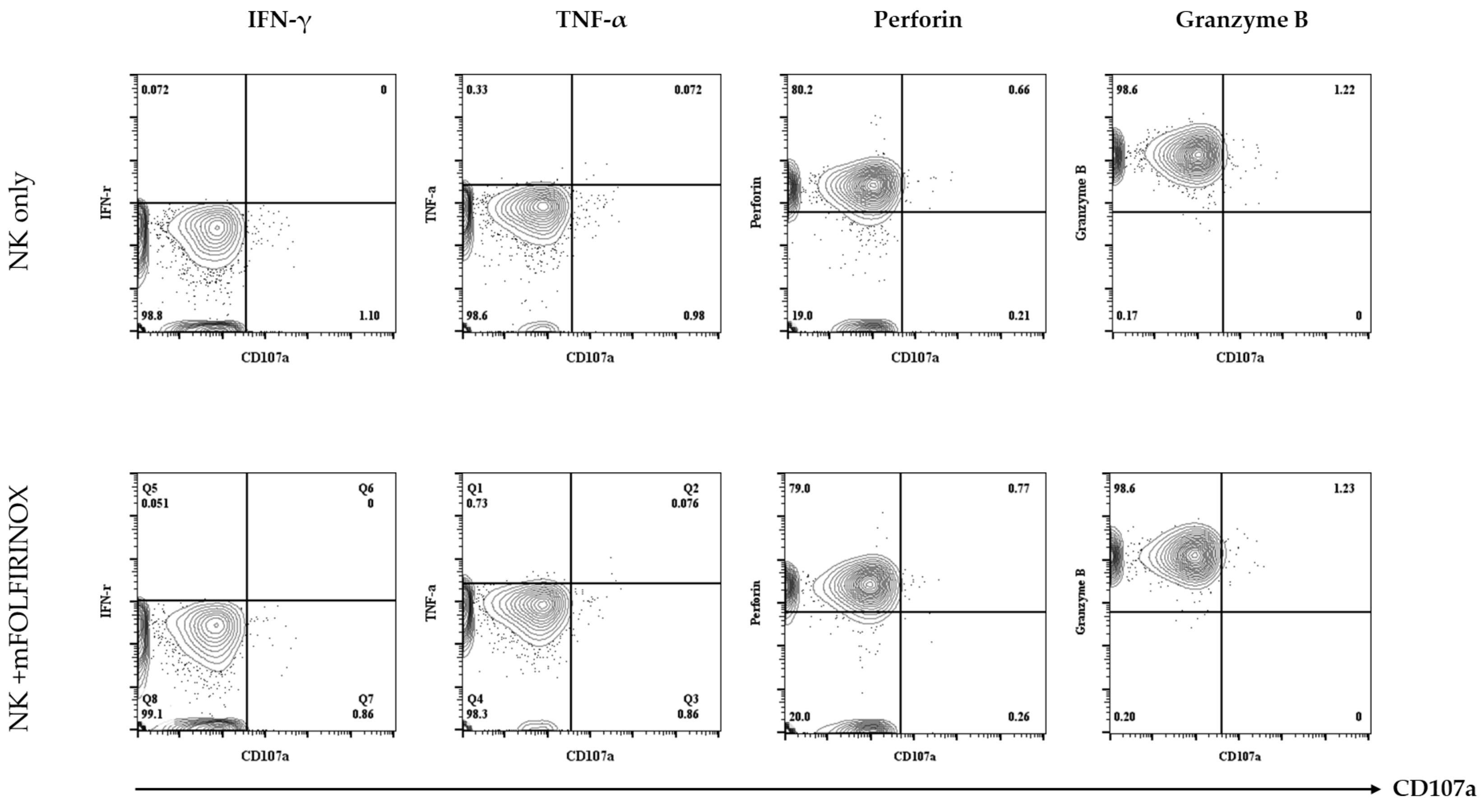


**Supplementary Figure S5.** Assessment of NK cell activation in response to direct mFOLFIRINOX treatment. NK cells were incubated alone (NK only, upper panels) or treated with mFOLFIRINOX (oxaliplatin 2.14 μM, irinotecan 2.56 μM, 5-fluorouracil 92.2 μM, and leucovorin 8.45 μM) for 24 hours (NK + mFOLFIRINOX, lower panels). Intracellular cytokines and cytotoxic molecules (IFN-γ, TNF-α, perforin, granzyme B) expression levels were measured by flow cytometry. The data illustrate that mFOLFIRINOX alone does not directly activate NK cells, as indicated by negligible changes in the expression of activation markers and effector molecules following treatment.

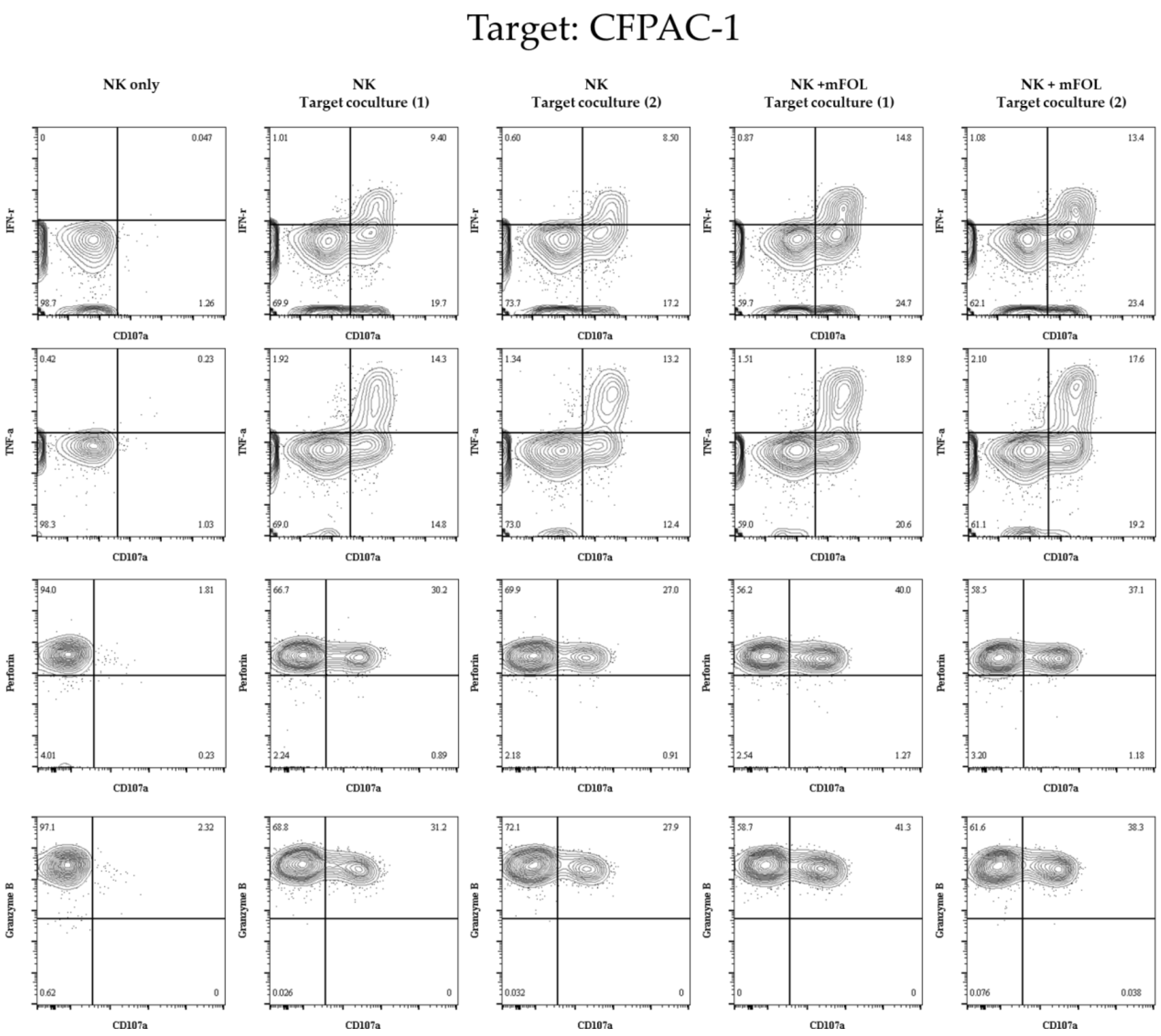


**Supplementary Figure S6.** Raw flow cytometry data corresponding to the functional response of NK cells co-cultured with CFPAC-1 pancreatic cancer cells. NK cells were cultured alone (NK only, first column), co-cultured with untreated CFPAC-1 cells (NK + Target co-culture, second and third columns), or co-cultured with CFPAC-1 cells pretreated with mFOLFIRINOX (oxaliplatin 2.14 μM, irinotecan 2.56 μM, 5-fluorouracil 92.2 μM, and leucovorin 8.45 μM; NK + mFOL Target co-culture, fourth and fifth columns). Each row represents flow cytometric analysis for specific NK cell markers and effector molecules: IFN-γ (first row), TNF-α (second row), perforin (third row), and granzyme B (fourth row). Horizontal axes indicate CD107a expression. Data illustrate representative plots used for the quantitative analysis presented in Figure 3B.

## Target: AsPC-1

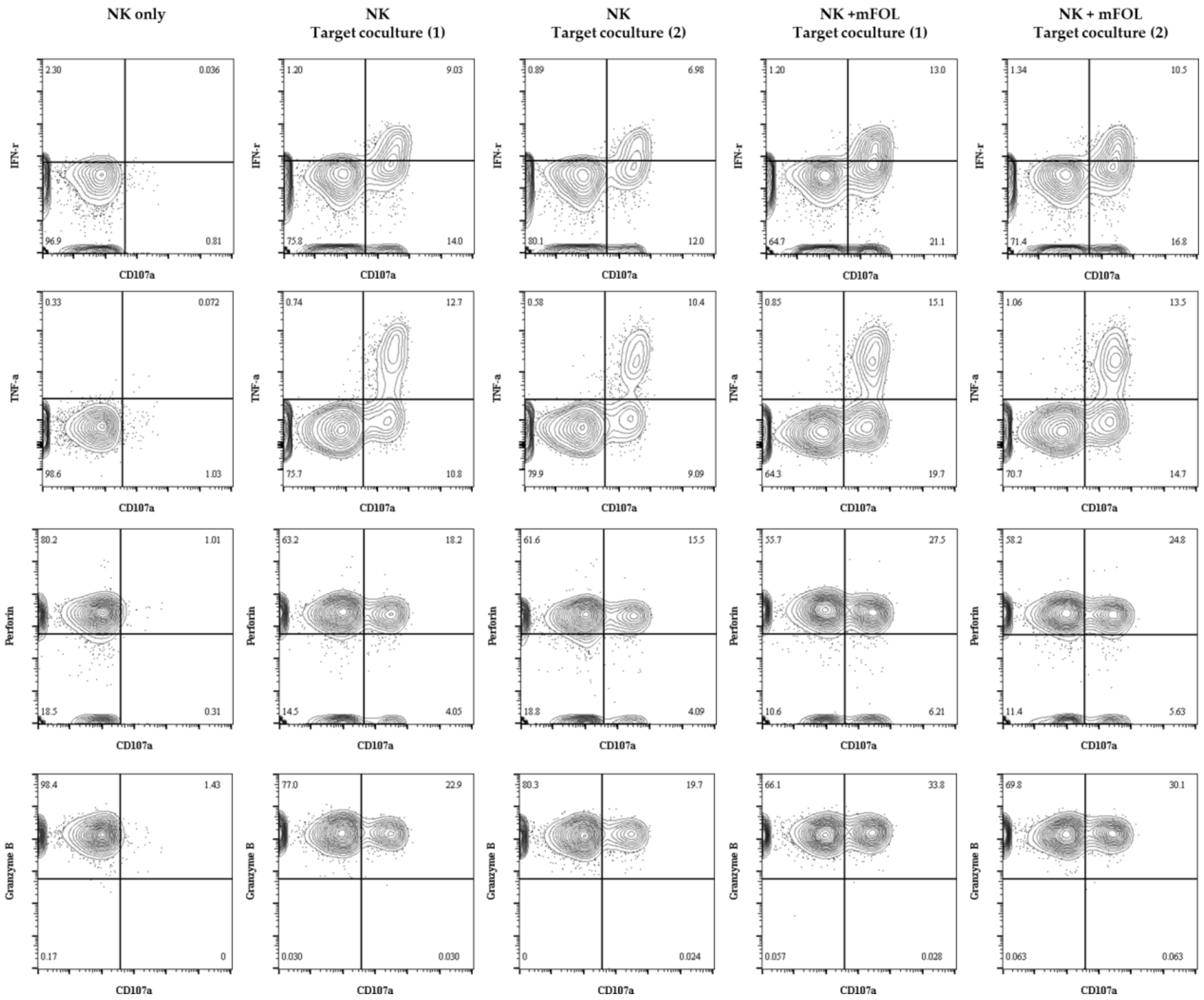


**Supplementary Figure S7.** Raw flow cytometry data corresponding to the functional response of NK cells co-cultured with AsPC-1 pancreatic cancer cells. NK cells were cultured alone (NK only, first column), co-cultured with untreated AsPC-1 cells (NK + Target co-culture, second and third columns), or co-cultured with AsPC-1 cells pretreated with mFOLFIRINOX (oxaliplatin 2.14 μM, irinotecan 2.56 μM, 5-fluorouracil 92.2 μM, and leucovorin 8.45 μM; NK + mFOL Target co-culture, fourth and fifth columns). Each row represents flow cytometric analysis for specific NK cell markers and effector molecules: IFN-$\gamma$ (first row), TNF-$\alpha$ (second row), perforin (third row), and granzyme B (fourth row). Horizontal axes indicate CD107a expression. Data illustrate representative plots used for the quantitative analysis presented in Figure 3B.

# Target: PANC-1

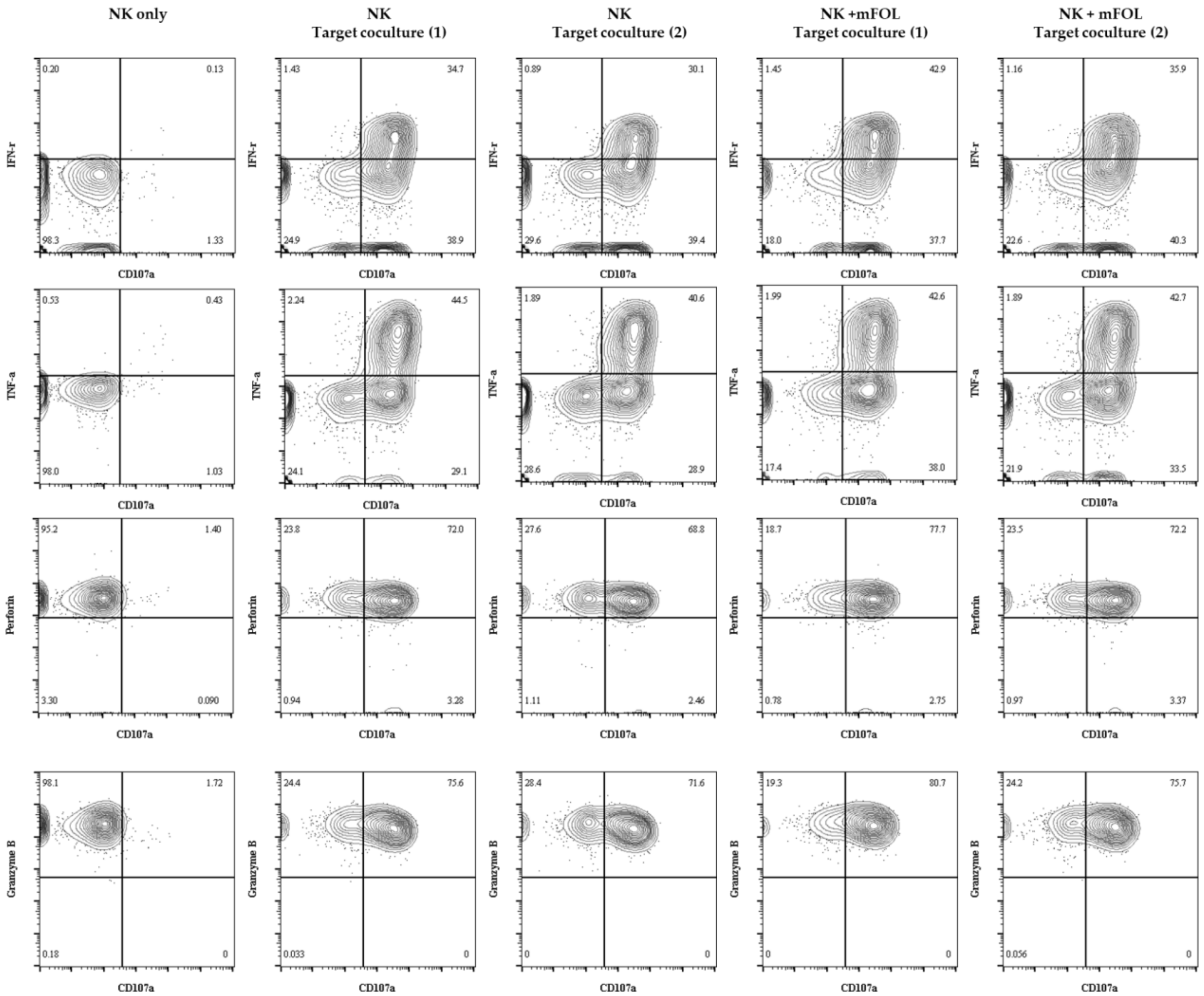


**Supplementary Figure S8.** Raw flow cytometry data corresponding to the functional response of NK cells co-cultured with PANC-1 pancreatic cancer cells. NK cells were cultured alone (NK only, first column), co-cultured with untreated PANC-1 cells (NK + Target co-culture, second and third columns), or co-cultured with PANC-1 cells pretreated with mFOLFIRINOX (oxaliplatin 2.14 μM, irinotecan 2.56 μM, 5-fluorouracil 92.2 μM, and leucovorin 8.45 μM; NK + mFOL Target co-culture, fourth and fifth columns). Each row represents flow cytometric analysis for specific NK cell markers and effector molecules: IFN-γ (first row), TNF-$\alpha$ (second row), perforin (third row), and granzyme B (fourth row). Horizontal axes indicate CD107a expression. Data illustrate representative plots used for the quantitative analysis presented in Figure 3B.

# mFOLFIRINOX Dose determination before in vivo Exp:

1. Literature based dose
2. Conversion based dose (Human dose → Mouse dose)

Literature-based dosing was selected with a specific focus on studies using NSG mice, rather than general preclinical models. This decision was made in light of PMID: 31409886, which highlighted that NSG mice exhibit significantly reduced tolerability to 5-FU compared to other strains.
This strain-specific sensitivity may also apply to other agents, which is why we focused exclusively on doses previously validated in NSG mice

1. Literature based dose
 1) Oxaliplatin: 7.5 mg/kg (PMID: 32661159)
 2) Irinotecan: 10 mg/kg (PMID: 27286453)
 3) 5′-FU: 20 mg/kg (PMID: 31409886)
 4) Leucovorin: 10mg/kg (PMID: 31409886)

Korean Clinical Practice Guidelines for Pancreatic Cancer (2021), pg88
Real practice of mFOLFIRINOX in CNUHH (the corresponding author's institution)
 1) Oxaliplatin: 85 mg/ $m^2$
 2) Irinotecan: 150 mg/ $m^2$
 3) 5′-FU: 1200 mg/ $m^2$
 4) Leucovorin: 400 mg/ $m^2$

**Table 1:** Animal Doses converted to Human Equivalent Doses Based on Body Surface Area*

| Species | Reference body weight (kg) | Working weight range (kg) | Body surface area ($m^2$) | To convert dose in mg/kg to dose in mg/ $m^2$, multiply by $k_m$ | To convert animal dose in mg/kg to HED in mg/ kg, either | |
|---|---|---|---|---|---|---|
| | | | | | Divide animal dose by | Multiply animal dose by |
| Human | 60 | - | 1.62 | 37 | - | - |
| Mouse | 0.02 | 0.011-0.034 | 0.007 | 3 | 12.3 | 0.081 |

Mohamed J. Saadh et al., *Sys Rev Pharm 2020;11(8):98-101*

 1) Oxaliplatin: 28.3 mg/ kg
 2) Irinotecan: 50 mg/ kg
 3) 5′-FU: 400 mg/ kg
 4) Leucovorin: 133.3mg/ kg

Dose reduction: ~4-fold for each drug..

2. Conversion based dose
 1) Oxaliplatin: 7 mg/kg (PMID: 32661159)
 2) Irinotecan: 12.5 mg/kg (PMID: 27286453)
 3) 5′-FU: 100 mg/kg (PMID: 31409886)
 4) Leucovorin: 33.3mg/kg (PMID: 31409886)

**Supplementary Figure S9.** Schematic diagram of the dose selection process for mFOLFIRINOX in NSG mouse models. To determine a tolerable and appropriate dose of mFOLFIRINOX prior to initiating in vivo studies, a dual approach was employed. First, literature-based doses were identified through an extensive review of previously published studies that administered mFOLFIRINOX in NSG mouse models. In parallel, clinical human doses were converted to mouse-equivalent doses using standard body surface area conversion formulas (conversion-based dose). Upon comparison, the conversion-based doses were found to be substantially higher than doses commonly used in prior NSG mouse studies. Considering potential toxicity at these higher doses, the conversion-based doses were conservatively reduced by approximately four-fold to establish the starting dose for initial in vivo experiments. The selected doses for the first experiment were as follows: for the literature-based dose, oxaliplatin 7.5 mg/kg, irinotecan 10 mg/kg, 5′-fluorouracil 20 mg/kg, and leucovorin 10 mg/kg; and for the conversion-based dose, oxaliplatin 7 mg/kg, irinotecan 12.5 mg/kg, 5′-fluorouracil 100 mg/kg, and leucovorin 33.3 mg/kg.

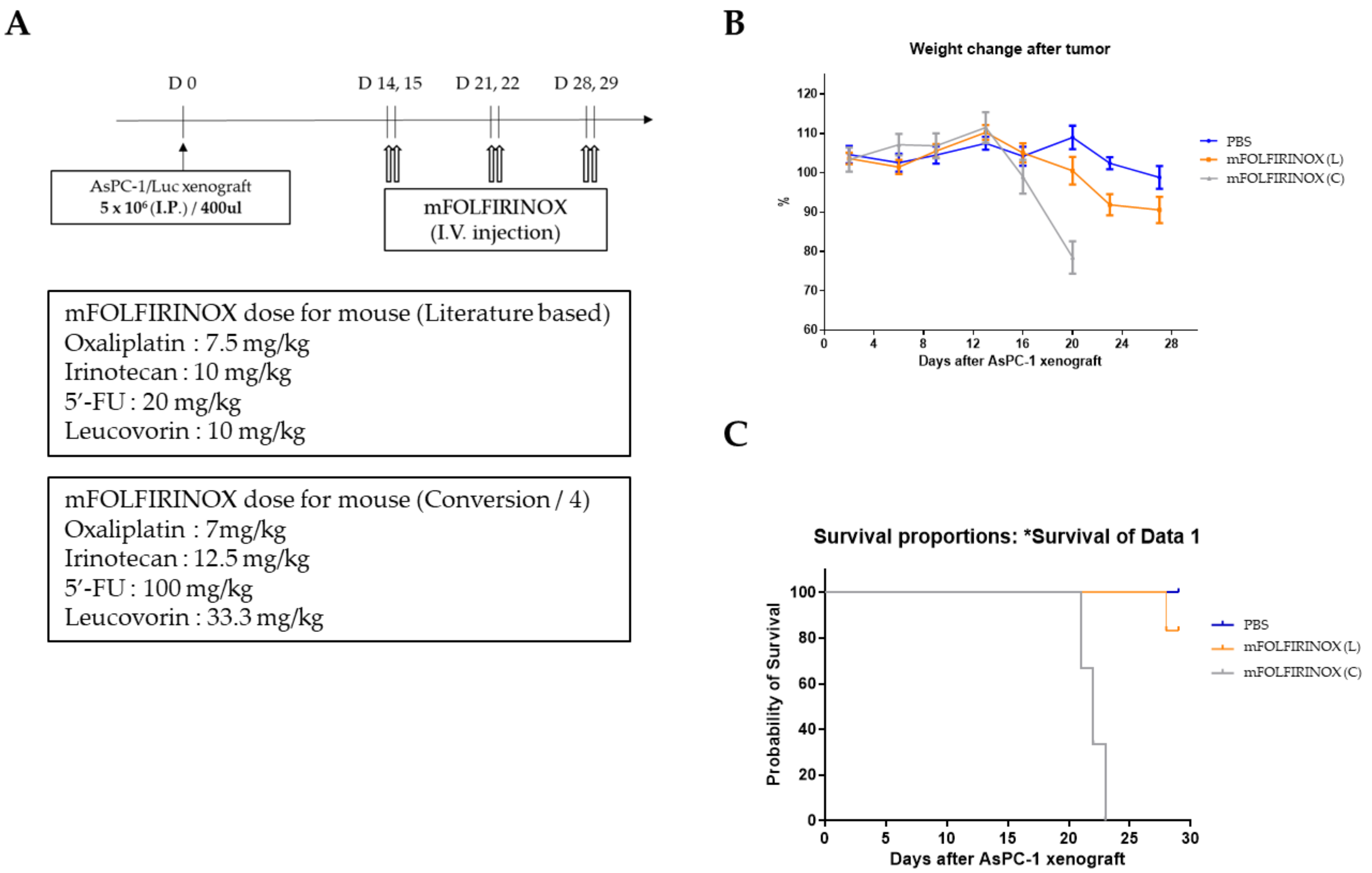


**Supplementary Figure S10.** Evaluation of tolerability and survival following mFOLFIRINOX treatment at different dose levels in a PDAC xenograft mouse model. (A) Schematic representation of the treatment regimen. AsPC-1/Luc cells (5 × 10⁶/400 μL) were injected intraperitoneally on Day 0. Beginning two weeks post-implantation, mice received mFOLFIRINOX intraperitoneally twice per week for three consecutive weeks. Two dose levels were tested: a literature-based dose (Oxaliplatin 7.5 mg/kg, Irinotecan 10 mg/kg, 5′-Fluorouracil 20 mg/kg, and Leucovorin 10 mg/kg) and a conversion-based dose reduced by four-fold (Oxaliplatin 7 mg/kg, Irinotecan 12.5 mg/kg, 5′-Fluorouracil 100 mg/kg, and Leucovorin 33.3 mg/kg). (B) Changes in body weight (%) were monitored throughout the study period to assess tolerability. (C) Kaplan-Meier survival curves of xenograft-bearing mice following treatment. The groups included PBS control (n = 6), mFOLFIRINOX literature-based dose [mFOLFIRINOX(L)] (n = 6), and mFOLFIRINOX conversion-based dose [mFOLFIRINOX(C)] (n = 3). .

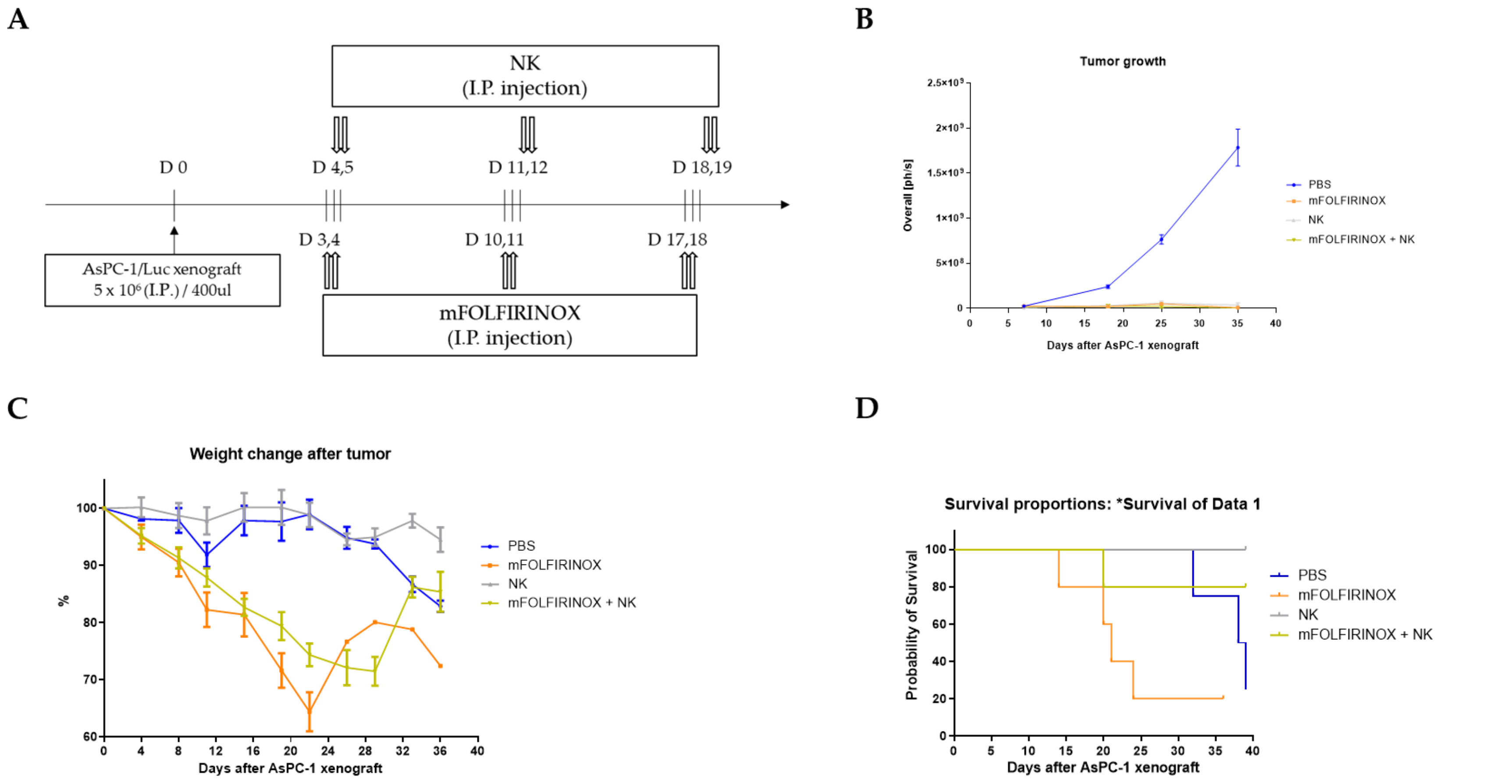


**Supplementary Figure S11.** Evaluation of the effects of mFOLFIRINOX in a PDAC mouse model. (A) Schematic representation of the in vivo treatment protocol. On Day 0, mice were injected intraperitoneally with $5 \times 10^6$ AsPC-1/Luc cells. Three days post-inoculation, mice were randomized into four groups: PBS control, mFOLFIRINOX, NK cells (NK), and mFOLFIRINOX combined with NK cells (mFOLFIRINOX+NK). Treatment was administered over three cycles at 1-week intervals. Each cycle consisted of mFOLFIRINOX administration on the first day [oxaliplatin 7.5 mg/kg, irinotecan 10 mg/kg, 5-fluorouracil 20 mg/kg, and leucovorin 10 mg/kg], followed by 5-fluorouracil alone (20 mg/kg) on the subsequent day. NK cells ($1.5 \times 10^7$/mouse) were administered intraperitoneally for two consecutive days, starting concurrently with 5-fluorouracil treatment. (B) Tumor burden was monitored weekly by bioluminescence imaging (BLI) and quantified as photons per second (ph/s). (C) Body weight changes (%) were recorded twice weekly throughout the study period to assess tolerability. (D) Kaplan-Meier survival curves were generated for each treatment group. The number of mice per group was as follows: PBS (n = 4), mFOLFIRINOX (n = 5), NK (n = 4), and mFOLFIRINOX+NK (n = 5).

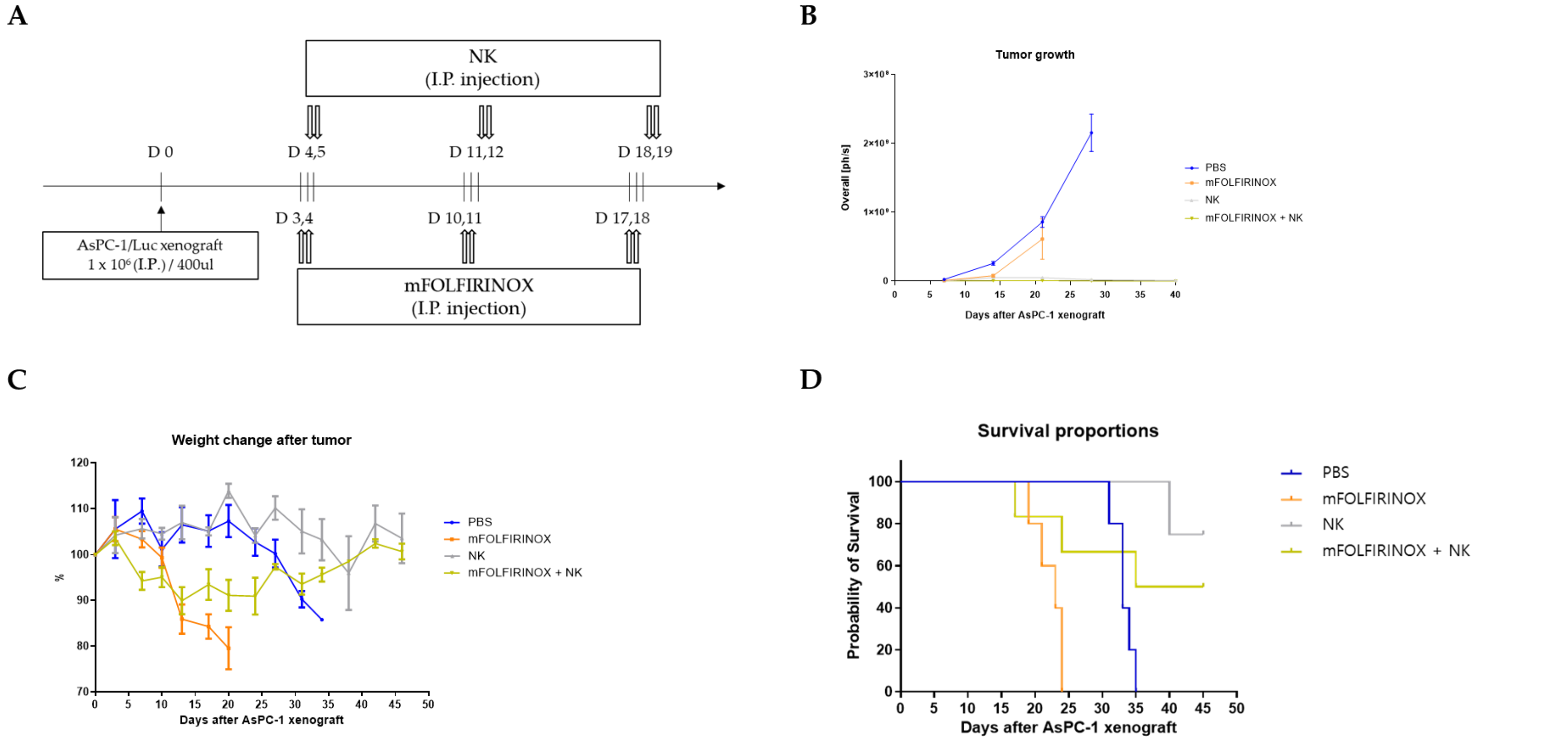


**Supplementary Figure S12.** Evaluation of the effects of dose-reduced mFOLFIRINOX in a PDAC mouse model. (A) Schematic representation of the in vivo treatment protocol. On Day 0, mice were injected intraperitoneally with $1 \times 10^6$ AsPC-1/Luc cells. Three days post-inoculation, mice were randomized into four groups: PBS control, mFOLFIRINOX, NK cells (NK), and mFOLFIRINOX combined with NK cells (mFOLFIRINOX+NK). Treatment was administered over three cycles at 1-week intervals. Each cycle consisted of mFOLFIRINOX administration on the first day [oxaliplatin 3.75 mg/kg, irinotecan 5 mg/kg, 5-fluorouracil 10 mg/kg, and leucovorin 5 mg/kg], followed by 5-fluorouracil alone (10 mg/kg) on the subsequent day. NK cells ($1.5 \times 10^7$/mouse) were administered intraperitoneally for two consecutive days, starting concurrently with 5-fluorouracil treatment. (B) Tumor burden was monitored weekly by bioluminescence imaging (BLI) and quantified as photons per second (ph/s). (C) Body weight changes (%) were recorded twice weekly throughout the study period to assess tolerability. (D) Kaplan-Meier survival curves were generated for each treatment group. The number of mice per group was as follows: PBS (n = 5), mFOLFIRINOX (n = 5), NK (n = 4), and mFOLFIRINOX+NK (n = 6).

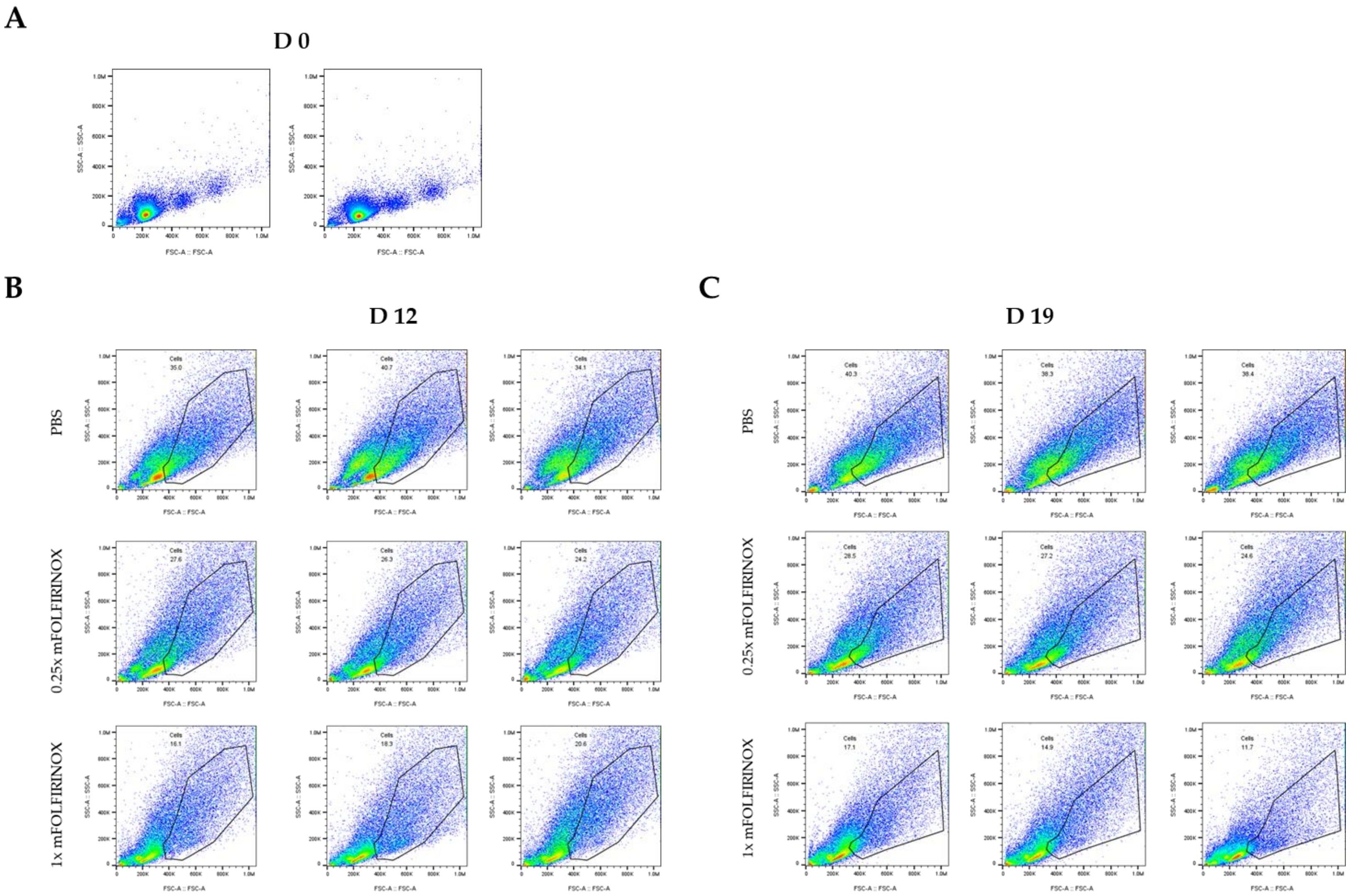


**Supplementary Figure S13.** Flow cytometric gating strategy for analysis of peritoneal cells following AsPC-1/Luc xenograft and mFOLFIRINOX treatment in vivo. (A) Representative FSC-A versus SSC-A dot plot of peritoneal lavage cells obtained from naïve (non-tumor-bearing) NSG mice on Day 0, demonstrating baseline peritoneal cell distribution. (B) FSC-A versus SSC-A plots of peritoneal lavage cells harvested from NSG mice 12 days after AsPC-1/Luc xenograft. Three treatment groups are shown: PBS (top row), 0.25× mFOLFIRINOX (middle row), and 1× mFOLFIRINOX (bottom row), with three mice per group (columns 1–3). The gated 'Cells' population was analyzed for the expression of NK cell-activating ligands and apoptosis-inducing receptors, corresponding to data presented in 'Cells' population was analyzed for ligand and receptor expression as shown in Figure 4E.ducing receptors (DR4, DR5, FAS) was subsFigure 4D. (C) FSC-A versus SSC-A plots of peritoneal lavage cells obtained from NSG mice 19 days post-xenograft, following the same treatment groups and layout as in (B). The gated equently analyzed within the gated live cell population.

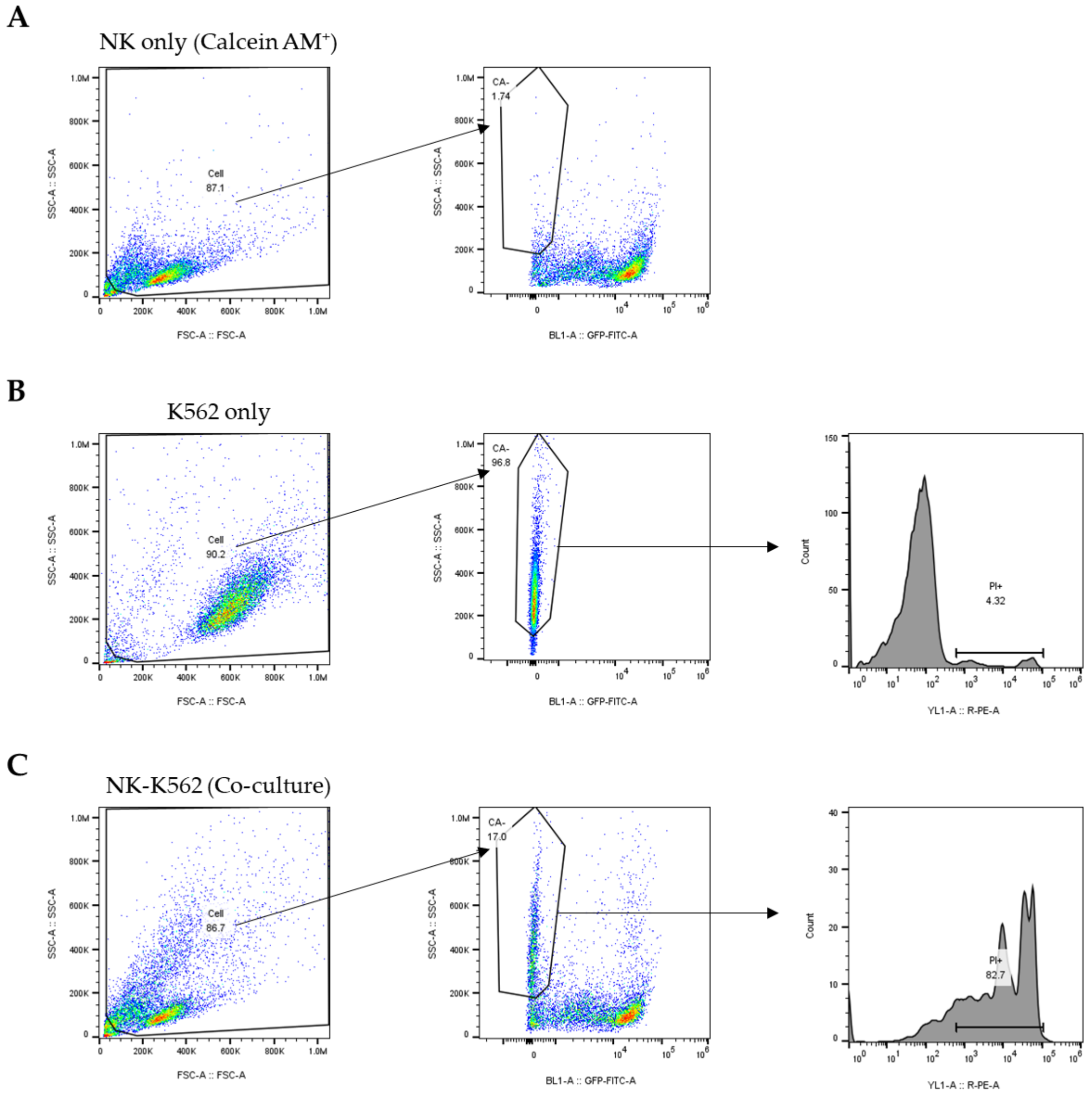


**Supplementary Figure S14.** Flow cytometry gating strategy for NK cell cytotoxicity assessment against K562 target cells (related to Table 2). To validate the cytotoxic function of expanded NK cells, a CFSE-based flow cytometry assay was performed. NK cells were labeled with CFSE to distinguish them from target K562 cells prior to co-culture. After a 4-hour co-culture at an effector-to-target (E:T) ratio of 4:1, propidium iodide (PI) staining was performed to identify dead target cells. (A) Flow cytometry plots showing FSC-A vs. SSC-A gating and CFSE positivity of NK cells (NK only control). (B) Gating strategy for K562 cells (K562 only control): FSC-A vs. SSC-A gating followed by selection of CFSE-negative (target, CA$^-$) population. The PI staining of CA$^-$ target cells is shown as a histogram; spontaneous PI$^+$ K562 cells accounted for 4.32%. (C) Gating strategy for NK-K562 co-culture: same sequential gating as in panel (B), followed by PI staining of CA$^-$ target cells to assess NK-mediated cytotoxicity. The increased proportion of PI-positive cells reflects NK-induced K562 killing. The proportion of CA$^-$PI$^+$ cells was used to calculate NK cytotoxicity, as presented in Table 2.

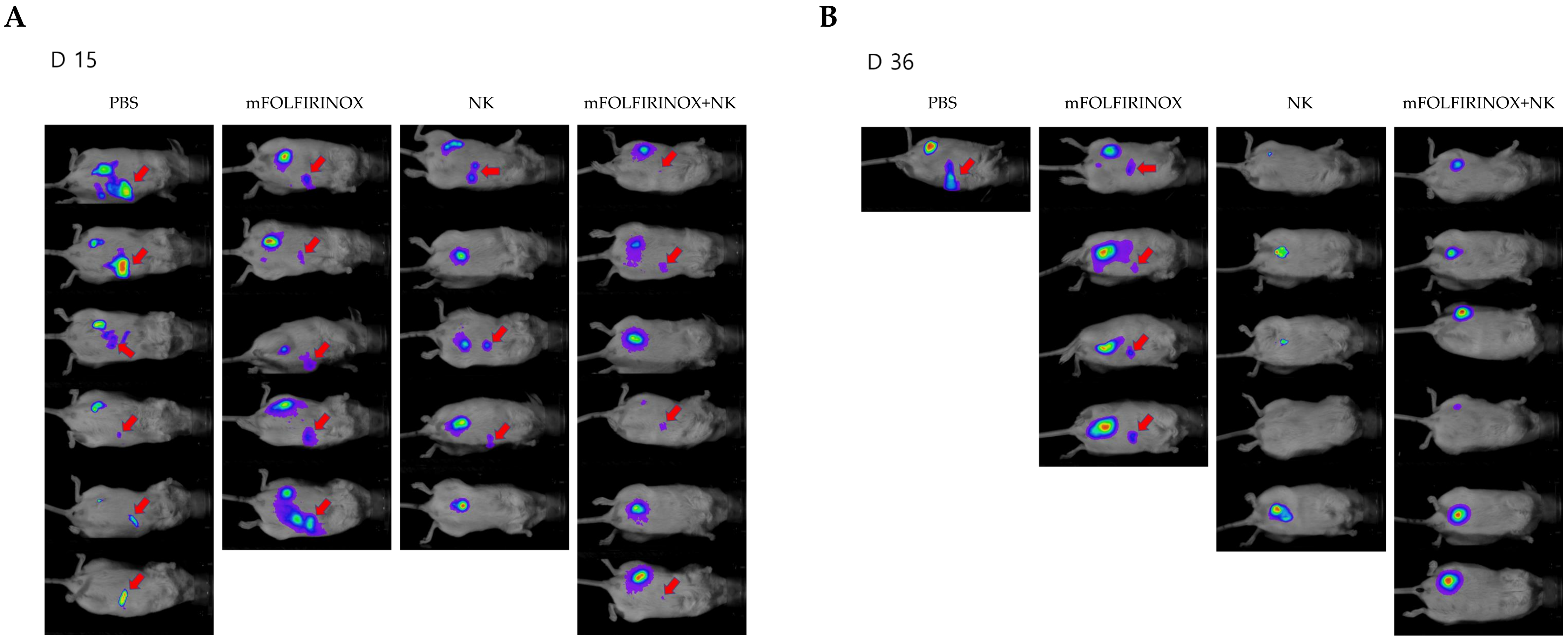


**Supplementary Figure S15.** Representative bioluminescence imaging (BLI) of tumor progression in the PDAC xenograft model, following the experimental design presented in Figure 5. NSG mice were intraperitoneally inoculated with 1 × $10^6$ AsPC-1/Luc cells (day 0). Treatment was conducted following the same experimental procedures as applied in Figure 5, and BLI images were presented to assess tumor burden at (A) day 15 and (B) day 36 post-tumor implantation. Ventral dorsal (VD) views are shown for each group: PBS control (column 1), mFOLFIRINOX (column 2), NK cells (NK; column 3), and mFOLFIRINOX combined with NK cells (mFOLFIRINOX+NK; column 4). Red arrows indicate localized bioluminescent signals near the pancreatic region. Photon flux scales were independently adjusted for each image to optimize the visualization of peripancreatic tumor signals.

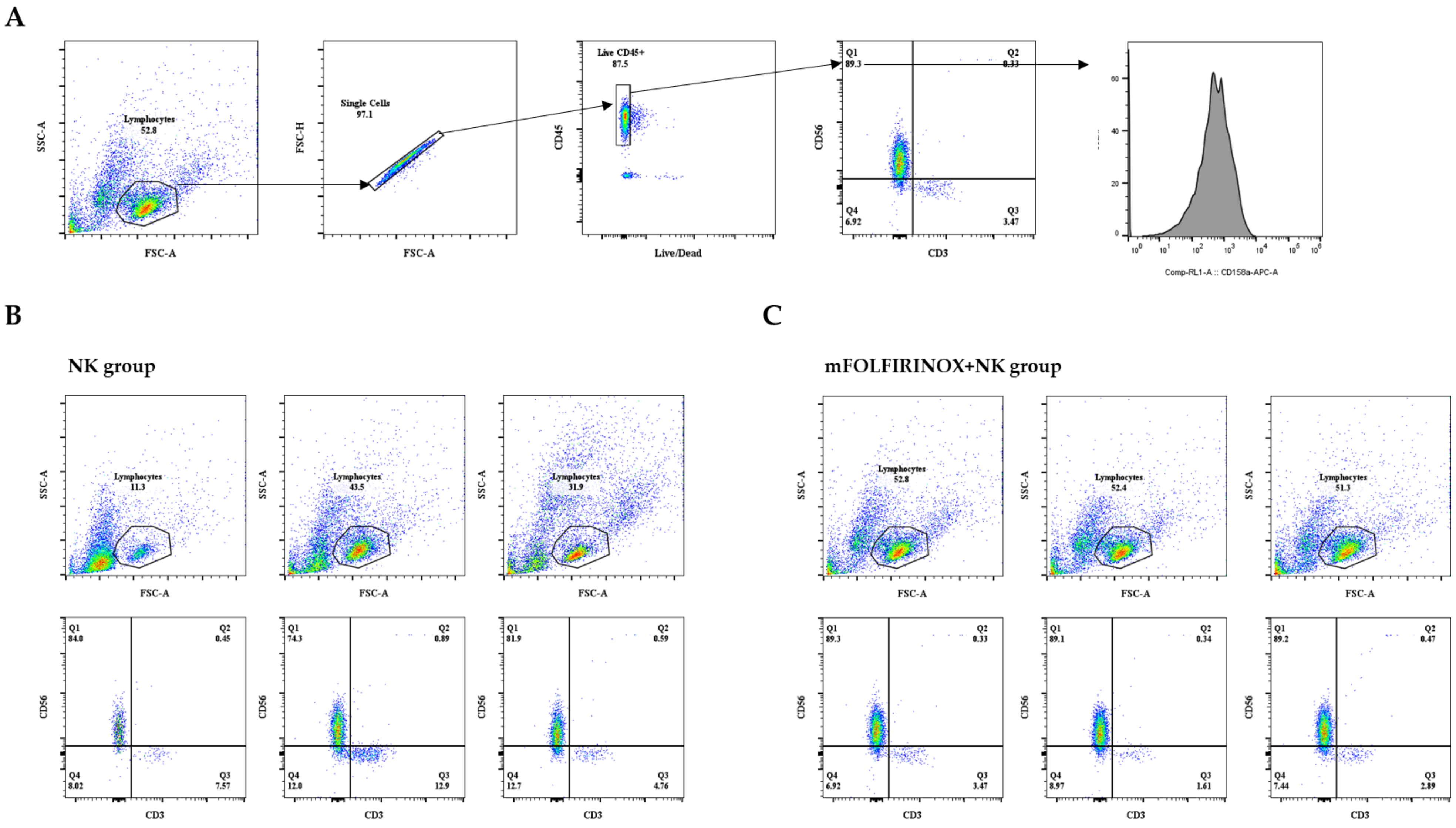


**Supplementary Figure S16.** Gating strategy and purity analysis of NK cells harvested from the peritoneal cavity in the PDAC xenograft model. (A) Flow cytometry gating strategy used to analyze NK cell purity and phenotypic markers following peritoneal lavage. Initial gating was performed on FSC-A vs. SSC-A to select cells, followed by singlet gating (FSC-H vs. FSC-A), live $CD45^{+}$ cell gating using viability dye, and subsequent CD3 vs. CD56 gating to identify $CD3^{-}CD56^{+}$ NK cell populations. The downstream phenotypic analyses correspond to Figures 6C and 6D. Representative plots from mice treated with NK cell monotherapy (NK group) (B) and combination therapy (mFOLFIRINOX+NK group) (C). The top rows show FSC-A vs. SSC-A plots from three individual mice. The bottom rows show corresponding CD3 vs. CD56 plots used to determine NK cell purity ($CD3^{-}CD56^{+}$ population), which are reflected in the purity analysis presented in Figure 6B.
